# Volcanic eruptions and the global subsea telecommunications network

**DOI:** 10.1007/s00445-025-01832-1

**Published:** 2025-06-04

**Authors:** Michael A. Clare, Isobel A. Yeo, Jacob Nash, James E. Hunt, Semisi Panuve, Alasdair Wilkie, Rebecca Williams, Natasha Dowey, Peter Rowley, Jennifer Barclay, Jeremy Phillips, Jazmin Scarlett, Samantha Engwell, Timothy J. Henstock, Sarah Seabrook, Sally Watson, Richard Wysoczanski, Marta Ribo, Shane Cronin, Peter J. Talling, Michael Cassidy, Sebastian Watt, Richard Robertson

**Affiliations:** 1 Marine Geoscience, National Oceanography Centre, Southampton, UK; 2 School of Ocean and Earth Science, University of Southampton, Southampton, UK; 3 Tonga Cable Ltd, Nuku’alofa, Tonga; 4 Digicel, Gros Islet, Saint Lucia; 5 School of Geography, Earth and Environmental Science, University of Hull, Hull, UK; 6 Department of the Natural and Built Environment, Sheffield Hallam University, Sheffield, UK; 7 School of Earth Sciences, University of Bristol, Bristol, UK; 8 Environment Agency, Bristol, UK; 9 British Geological Survey, Nottingham, UK; 10 New Zealand Institute of Water and Atmospheric Science, Aotearoa, New Zealand; 11 Auckland University of Technology, Aotearoa, New Zealand; 12 University of Auckland, Aotearoa, New Zealand; 13 Schools of Geography and Earth Science, Durham University, Durham, UK; 14 Birmingham University, Birmingham, UK; 15 The University of West Indies Seismic Research Centre, St. Augustine, Saint George, Trinidad and Tobago

**Keywords:** Volcanic eruption, Submarine cables, Telecommunications infrastructure, Volcanic hazards, Marine geohazards

## Abstract

When the first transoceanic telegraph cables were laid in the mid-1800s, rapid communication between continents became possible. The advent of fibre-optic submarine cables in the 1990s catalyzed a global digital revolution. Today, a network of > 1.7 million kilometres of fibre-optic cables crosses the oceans, carrying more than 99% of all digital data traffic worldwide and trillions of dollars in financial transactions. These arteries of the global internet underpin many aspects of our daily lives, and are particularly important for remote island communities that rely on submarine cables for telemedicine, e-commerce, and online education. However, these same remote communities are often in seismically and volcanically active regions and can be prone to natural hazards that threaten their critical subsea communication infrastructure. This vulnerability was acutely exposed in January 2022, when the collapse of the eruption plume of Hunga Volcano triggered fast-moving density currents that damaged Tonga’s only international submarine cable, cutting off an entire nation from global communications in the midst of a volcanic crisis. Here, we present a new comprehensive analysis of damage to subsea communications cables by volcanic events from around the world, and document their diverse impacts. Examples include (i) severing of the telegraph cable crossing the Sunda Strait by a tsunami triggered by the 1883 Krakatau eruption, Indonesia; (ii) ocean-entering pyroclastic density currents, lahars, and landslides during the 1902 eruptions of Mount Pelée, Martinique, that damaged six telegraph cables; (iii) destruction of a cable landing station on Montserrat by a pyroclastic density current in 1997; (iv) submarine slope failure at Kick ‘em Jenny, Grenada, that damaged two fibre-optic cables; (v) complete loss of the telecommunications network due to power outages following the 2000 eruption of Miyake-jima, Japan; and (vi) disruption to subsea cables resulting from the 2021 eruption of La Soufrière, St. Vincent. We find that the causes of damage typically relate to secondary hazards that occur not only at the same time as the eruption climax, but also some time after. There does not appear to be an explosivity intensity threshold for cable-damaging events; however, the extent of damage may be related to the original volcano morphology (e.g. steep slopes), spatial location (e.g. near the coast or partially/totally submerged), the eruption size or explosivity, and/or volcanic depositional processes involved. Based on these diverse case studies, we present lessons learned for enhancing telecommunications resilience, and discuss how subsea cables themselves can be used as sensors to improve understanding and early warning of volcanic hazards, potentially filling a monitoring gap for remote island communities.

**Supplementary Information:**

The online version contains supplementary material available at 10.1007/s00445-025-01832-1.

## Introduction

Hazardous phenomena resulting directly and indirectly from volcanic eruptions can threaten all types of infrastructure, which can hinder effective disaster response in the immediate aftermath, as well as having longer-lasting economic impacts. Numerous processes during and following volcanic eruptions, including ash fall, pyroclastic density currents (PDCs), lava flows, and lahars, are responsible for a variety of impacts to infrastructure. Damage to critical terrestrial lifelines has been well demonstrated, including impacts on electrical supplies, drinking water, sewerage, and filtration systems, along with communications and transportation networks (e.g. Bebbington et al. 2008; Jenkins et al. 2014; Sword-Daniels et al. 2014; Wilson et al. 2014 Hayes et al. 2019; Williams et al. 2020; Salgado et al. 2023; Santos et al. 2023). Elements of terrestrial infrastructure can be damaged directly, or by a set of cascading impacts, such as power, communications, or ancillary equipment outages. Post-eruptive processes may extend the adverse effects of eruptive activity, re-damaging repaired infrastructure months to years after the climax of an eruption (e.g. Pierson et al. 2013; Phillips et al. 2024).

### A motivation to assess threats posed to subsea telecommunications networks

While several studies have addressed the impacts of volcanic hazards on terrestrial telecommunications infrastructure (Table 1), it was not until the recent eruption of Hunga volcano (formerly known as Hunga Tonga-Hunga Ha’apai) that wider attention has been placed on these hazards for subsea networks. The eruption of Hunga volcano in January 2022 (Kingdom of Tonga) was the most explosive eruption of any submerged volcano in > 100 years (Volcanic Explosivity Index (VEI) of 5–6; Newhall and Self 1982; Borerro et al., 2023). The eruption had widespread impacts including ash fall that affected onshore telecommunication and power networks, tsunamis that caused severe damage to onshore infrastructure (Lynett et al. 2022; Pakoksung et al. 2022; Borrero et al. 2023), and an eruption column that reached a height of 57 km above sea level, spreading to a diameter of > 600 km, and limiting satellite communications in the immediate aftermath (Carr et al. 2022; Proud et al. 2022). While these terrestrial impacts are undoubtedly important, one of the most profound impacts was felt under the sea. Powerful sediment-laden seafloor flows were triggered when large volumes of pyroclastic material plunged into the ocean as the eruption column collapsed (Clare et al. 2023a). These seafloor flows, which travelled at speeds of up to 122 km/h, devastated the biology in their path and damaged the two subsea telecommunications cables that connected to the Kingdom of Tonga, including the sole international cable that provided connection to the wider global network via Fiji (Clare et al. 2023a; Seabrook et al. 2023). When the international cable was cut, this disconnected Tonga from global digital communications at a critical time for disaster response.

**Table 1:** Examples of impacts of volcanic eruptions on land-based telecommunications infrastructure

Nature of impact	Implications for terrestrial communications	Documented examples of impacts
Direct	Attenuation or reduction of the signal strength of radio and electromagnetic broadcasts	Due to influence of electrically charged ash particles within the ash cloud reported during the 1991 Pinatubo eruption, Philippines (Wilson et al. 2014)
	Direct damage to telecommunications equipment by ash fall	Shut down of Anchorage telephone exchange during the 1992 Crater Peak eruption, Alaska (USA) when ash fall blocked cooling systems (Wilson et al. 2012)
Indirect	Overloading of telecommunications networks due to high user demand during a volcanic eruption	Excessive use of telecommunications led to temporary network shutdown during 2008 eruption of Chaitén volcano, Chile (Wilson et al. 2012)
	Damage to other infrastructure upon which telecommunications systems rely, such as power supplies	Damage of electricity transmission network leading to shutdown of telecommunications in 1995 due to the impacts of pyroclastic density currents, lahars, and ash fall from the eruption of Soufrière Hills volcano, Montserrat (Wilson et al. 2014). Conductive wet ash coated electrical networks, leading to power outages around Mount Ruapehu, New Zealand, in 1995–1996 (Johnston et al. 2000)

**Fig. 1:**
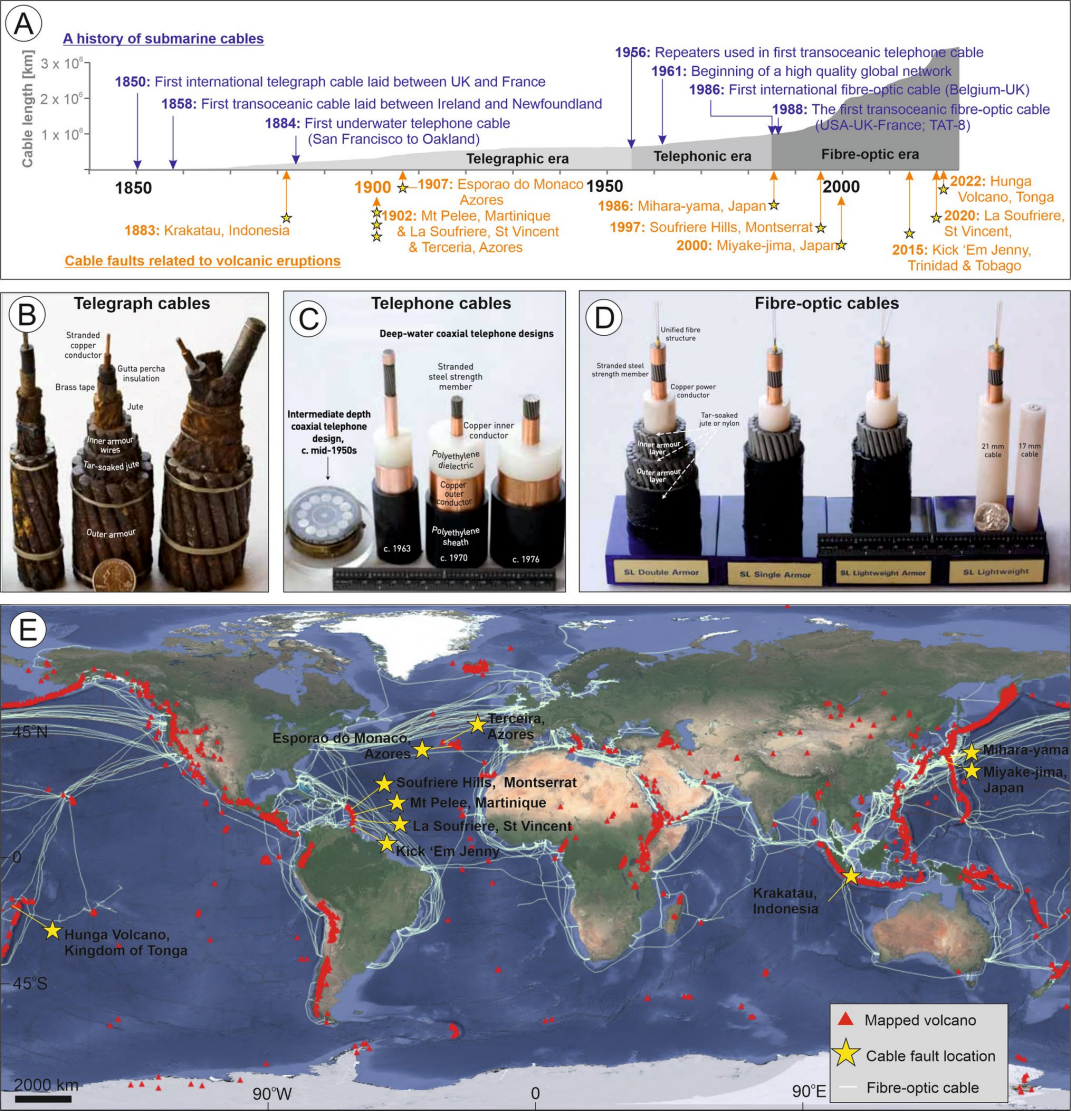
Overview of case studies in this review and their timing and location relative to the expansion of the subsea cable network.** A** Timeline illustrating the history of subsea cables (blue) and cable damage associated with volcanic eruptions (orange). Instances of cable damage are annotated by yellow stars. Photographs show examples of cables from the telegraph (**B**), telephonic (**C**), and fibre-optic (**D**) era.** E** Geographic distribution of in-service fibre-optic subsea cables, cable faults (stars), and volcanoes (red triangles) based on GVP (2024). Background relief is from Google Earth

The Hunga volcano shows that eruptions can have significant impacts under the sea, yet comparatively little attention has been paid to the consequences of volcanic activity on offshore infrastructure relative to those onshore. Our daily lives rely upon digital communications, from emails, video messaging, remote working, internet banking, and e-commerce to social media, yet many people are unaware that > 99% of all digital data traffic worldwide is carried by submarine telecommunications cables, due to bandwidth limitations of satellites (Telegeography 2023). Presently, a network of more than 1.7 million km of in-service fibre-optic telecommunications cables crosses the seafloor worldwide, underpinning the internet and carrying trillions of dollars a day in financial transactions (Fig. 1; Clare et al. 2023b). Remote island nations, such as Tonga and others in the South Pacific, particularly rely upon these cabled links for remote access to online education, telemedicine, tourism bookings, and for key financial transactions (Carter 2009). For example, around 40% of Tonga’s Gross Domestic Product comes from remittances (i.e. funds sent from abroad; World Bank 2023). When Tonga’s only international cable was cut shortly after the 2022 eruption of Hunga volcano, its primary income stream stopped, businesses could no longer transact, and the country was cut off from international communications.

### A brief history of the global subsea telecommunications network

Our reliance on subsea cables is far from new, although it has increased markedly in recent years. Global communications were transformed when subsea telegraph cables first connected countries together. The first international telegraph cable was laid between the UK and France in 1850, with the first transoceanic cable laid soon after between Ireland and Newfoundland, Canada, in 1858 (Fig. 1; Carter 2009). Before that time, communications between Europe and North America relied on ships, which were often delayed by bad weather. The revolutionary new connection by subsea cable allowed for telegrams to be transmitted and replied to within the same day. Rapid expansion of subsea telegraph cables led to a global network, with all the world’s populated continents connected when Java was connected to Australia in 1871 (Moyal 1983). These telegraph cables had a multi-stranded copper wire at the core, initially wrapped in a natural latex (gutta percha) to be replaced later by polyethylene for insulation, and cased in iron or steel wire for protection (Fig. 1B). The next major leap forward was the development of co-axial cables that enabled transmission of telephone communications, and repeaters that boosted the signal enabling their use in trans-oceanic systems (Carter 2009). The first trans-oceanic telephone cable system (TAT-1; Transatlantic No. 1) connected Scotland and Newfoundland in 1956, carrying 36 telephone channels (Fig. 1C; Kelly et al. 1955). By 1961, a high-quality global telephone network was established; however, it would not be long before technological advances progressed further, with the development of fibre-optic cables. The first trans-oceanic fibre-optic cable was laid between the UK, France, and the USA (TAT-8) in 1988 with many more systems laid over the following years, particularly in the dot-com boom of the mid-1990 s (Carter 2009). Such cables, which are typically no wider than a garden hose (17–22-mm diameter for most deep-sea cables; Fig. 1D), enable low-latency and high-bandwidth digital communications, carrying terabits per second (compared to megabits per second for satellites) and form the basis of the modern global network of subsea cables (Fig. 1; Carter 2009; Clare et al. 2023b). The total length of subsea telecommunications cables (of all types, including telegraph, telephonic, and fibre optic) to have been installed to date is now estimated at > 3.5 million km (Fig. 1A; Appleby and Dawe 2019). When cables are laid in less than 1000 m of water depth, they are typically buried for their protection (Clare et al., 2023a), but this is not always possible in volcanic settings due to the highly competent nature of bedrock that outcrops at the seafloor, sometimes highly irregular topography, and variable depths or local absence of sedimentary cover (Mitchell et al. 2002).

### Factors that cause damage to subsea cables

While the existing, and ever-expanding, global network of subsea telecommunications cables is remarkably resilient, there are 150–200 instances of damage every year that require repair (Kordahi et al. 2019; Bricheno et al. 2024). Most damage occurs in relatively shallow water (< 200 m), relating to accidental human activities (e.g. entanglement with fishing gear, anchor drops from ships). Despite accounting for a relatively small proportion of damage events (10–20%), natural hazards associated with underwater landslides, earthquakes, volcanic eruptions, and tropical storms are particularly significant. This is because their large hazard footprint may synchronously damage multiple cable systems from shallow to deep waters. This can limit re-routing of data traffic, can cut off entire regions or countries, and lead to repairs that can reach hundreds of millions of dollars and that can amount to far larger costs due to loss of data traffic and financial trading (Carter et al. 2014). This situation was exemplified in early 2022 at Hunga volcano, where extensive cable damage occurred in deep water as far as 80 km away from the volcano. Some studies have assessed the threats posed to subsea telecommunications cables by a range of natural hazards (e.g. earthquakes, Pope et al. 2017a; tropical cyclones, Gavey et al. 2017, Pope et al. 2017b; river floods and sediment flows, Talling et al. 2022; climate change hazards, Clare et al. 2023c); however, to date, none has focused on the impacts of volcanic activity. While relatively rare, compared to other natural hazards, cable damage can be extensive with wide-reaching implications. Here, we address this knowledge gap through analysis of a first global timeline of subsea cable damage by volcanic eruptions.

## Aims

In this study, our overarching aims are to determine the following: (i) to what extent volcanic eruptions pose a threat to subsea cables; (ii) what we can learn about the different types of submarine hazards from instances of cable damage; and (iii) how to enhance the resilience of telecommunications connections in volcanically active settings. Specifically, we address the following questions. First, whether the 2022 eruption of Hunga volcano was an exception, or if there have there been other instances of damage to subsea cables from volcanic eruptions. We present a time series based on new analysis of a database of cable damage and a literature review to identify at least 11 eruptions that caused damage to multiple subsea cables at numerous locations worldwide. Second, when during an eruption sequence does cable damage occur and which process(es) are the most damaging? We present case studies from around the world to investigate whether cable damage occurs consistently at the climax (i.e. most intense part) of each eruption; if not, then what aspect of the eruption sequence is responsible, and to what extent this varies between volcanoes. Third, we assess the type of eruption that results in cable damage, looking at whether all eruptions close to subsea cables have resulted in damage. Specifically, we explore whether there is a VEI threshold above which cables are likely to be damaged. Observations of cable damage can provide unique observations of the offshore nature and extent of volcanic eruptions that may otherwise be missed. Hence, fourth, we ask what these instances of cable damage reveal about the fundamental behaviour and evolution of volcanic processes that enter, or initiate within the ocean? Finally, we conclude with the lessons that can be learned for enhancing resilience of subsea cable networks in active volcanic terrains. We discuss how, and to what extent, the impacts of volcanic hazards can be mitigated.

## Data and methods

This study includes two aspects. The first is presented as a series of case studies that are developed from collating available information about the timing and location of cable breaks— either provided by the cable owner, from a proprietary database shared with us by OceanIQ Ltd, or determined from reports, written accounts, and literature in the public domain. For each case study, we also synthesise a time series to include the onset, climax, and end of an eruptive episode within which there was a reported cable break or breaks (Tables S-2 to S-6). These time series are based on existing published literature and written accounts collated by the authors, and include new data gathered from evidence of cable damage offshore La Soufrière, St Vincent in 2021, and Kick ‘em Jenny volcano, offshore Grenada in 2015. The primary sources of information range from eyewitness accounts, evidence of morphologic change determined from satellite and offshore surveys, to real-time monitoring using land-based and seafloor seismometers, river gauges, and satellite imaging. The second aspect places the case studies in a broader global context. We use the Global Volcanism Program (GVP) database (GVP, 2024), which includes volcanic eruptions known to have occurred over the past 12,000 years. This database is the source for the VEI stated in each of the case studies. The VEI describes the size of explosive volcanic eruptions based on magnitude and intensity and ranges from VEI 0 (non-explosive) to VEI 8 on a logarithmic scale (Newhall and Self 1982). It is important to note that for many subaerial eruptions the assigned VEI remains unverified, and it is particularly challenging to quantify for many submarine eruptions. We use the GVP database to identify the locations of eruptions that did not result in cable damage, as well as to identify volcanoes that may feature potentially cable-damaging eruptions, but that erupted during a time window before a cable was installed. The straight-line distance from volcanoes to subsea cables was determined from a topo-bathymetric digital elevation model using the Find Nearest tool in ArcGIS. We did not screen the results of the GVP database, other than retaining those that occurred during the time window during which cables have been in use. The impacts of volcanic eruptions referenced in the following text, but which do not form the basis of specific case studies, are summarised in supplementary Table S-1.

## Results

### Overview of damage to subsea cables by volcanic hazards worldwide

Our analysis shows that there have been at least 11 eruptions since the installation of subsea cables in the 1850s that have led to damage to one or more subsea cables worldwide (Fig. 1A, E; Table 2). These events relate to eruptions at volcanic islands (*N* = 7) or underwater volcanic edifices (*N* = 4), and are particularly significant compared to typical cable damage for a number of reasons. The first is that eight out of 11 volcanic events damaged the primary and only cable, disconnecting an island or region. Second, some volcanic events damaged multiple cables; in some cases, up to six cables were damaged by a single event. Third, the extent of the damage is greater than that experienced during typical instances of cable breaks due to bottom contact fishing or anchor drops, which tend to be very localised. While the full extent of damage is not known for all cases, evidence from cable repairs reveals that cable lengths of 10–100 km can be damaged or buried beyond recovery. Additionally, cable damage can occur in a wide range of water depths, from shallow coastal (tens of metres) to at least 2.4 km depth, and in some cases tens of kilometres away from the volcano. In two cases, the land-based cable station was affected by volcanic activity rather than the subsea cable, but this effectively stopped connection to the cable, so the same impacts were felt.

**Table 2:** Overview of instances of damage to subsea cables associated with volcanic eruptions, referencing explosivity (VEI), extent of damage, proximity of nearest cable to the volcano, and the interpreted cause of damage where known. Note that precise date of damage is not known for all events; hence, in some cases only the year is noted

Date of cable damage^1^	Volcano (VEI)	Location	Water depth of damage	Extent of damage	Interpreted cause of damage
26 August 1883	Krakatau (VEI 6)	Indonesia, Indian Ocean	< 50 m	Damage to Sunda Strait telegraph cable coming into Anjer, Java. Extent unknown	Damaged by vessel sunk by tsunami
8 May 1902	Mount Pelée (VEI 4)	Martinique, Caribbean	Likely up to 2000 m	5 of 6 telegraph cables damaged up to 26 km from shore	Multiple causes including ocean-entering lahars, pyroclastic density currents and submerged slope failure
7 May 1902	La Soufrière (VEI 4)	St Vincent, Caribbean	Likely up to 2000 m	All 6 telegraph cables damaged 900 m of vertical seafloor change (loss)	Precise timing only known for one cable, interpreted to be due to slope failure triggered by lahars and due to ocean-entering pyroclastic density currents
7–8 May 1902	Submarine volcano, West of Terceira (VEI unknown)	Azores, North Atlantic	Between 450 and 1400 m	Only telegraph cable damaged. Various ruptures along 16-km length	Cause unknown — assumed slope failure
1 April 1907	Esporao do Monaco submarine volcano (VEI unknown)	Azores, North Atlantic	400 m	Only telegraph cable damaged, buried in fine-grained volcanic deposits	Cause unknown — assumed slope failure
1986	Mihara-yama, O-shima (VEI 0)	Japan, North Pacific	N/A	Temporary outage on only coaxial cable due to brief loss of power supply	Earthquake caused loss of power supply
1997	Soufrière Hills (VEI 3)	Montserrat, Caribbean	N/A	Cable landing station completely destroyed, cutting the only fibre-optic cable connection	Pyroclastic density current
2000	Oyama, Miyake-jima (VEI 0)	Japan, North Pacific	N/A	Telecommunications suspended on only fibre-optic cable	Power outage due to evacuation of the island due to hazardous volcanic gas emissions
23 July 2015	Kick ‘em Jenny (VEI 0)	Grenada, Caribbean	2020 and 2430 m	The only two fibre-optic cables were damaged, located 12 km and 18 km from Kick ‘Em Jenny	Submarine debris flows originating from the crater rim
9 April 2021	La Soufrière (VEI 1–4)	St Vincent, Caribbean	1100–2500 m	Two fibre-optic cables damaged (one along 20 km, another along 24-km length), up to 20 km from the island	Slope failure possibly associated with ocean-entering lahars
15 January 2022	Hunga (VEI 5–6)	Kingdom of Tonga, South Pacific	Up to 1800 m	The only two fibre-optic cables were damaged along 89-km (international) and 105-km (domestic) lengths	Volcaniclastic density currents triggered by eruption column collapse

### Examples of subsea cable damage associated with volcanic eruptions

Our analysis reveals that the volcano-related processes that cause cable damage differ between eruptions and locations; hence, we now discuss each of these events as individual case studies. We determine when in the eruption sequence the cable damage occurred and how confidently we can attribute a causative process and mechanism to the damage. In the following, we use *eruption episode* to signify the whole period of volcanic unrest, and the eruption, which may last for hours to months, depending on the situation; this can be synonymous with *eruption* when discussing the episode in general terms or in summary. We use the term *climactic phase* to refer to the period within the episode with the greatest intensity or mass-eruption rate. Eruption *phase* refers to a period of time when a particular type of activity dominates (e.g. a phase of lava effusion or dome emission, followed by a phase of explosive activity). An *event* is used to denote a particularly distinctive happening during an eruption, for example, collapse of a flank, or a particularly large explosion.

#### Krakatau, Indonesia, August 1883

**Fig. 2:**
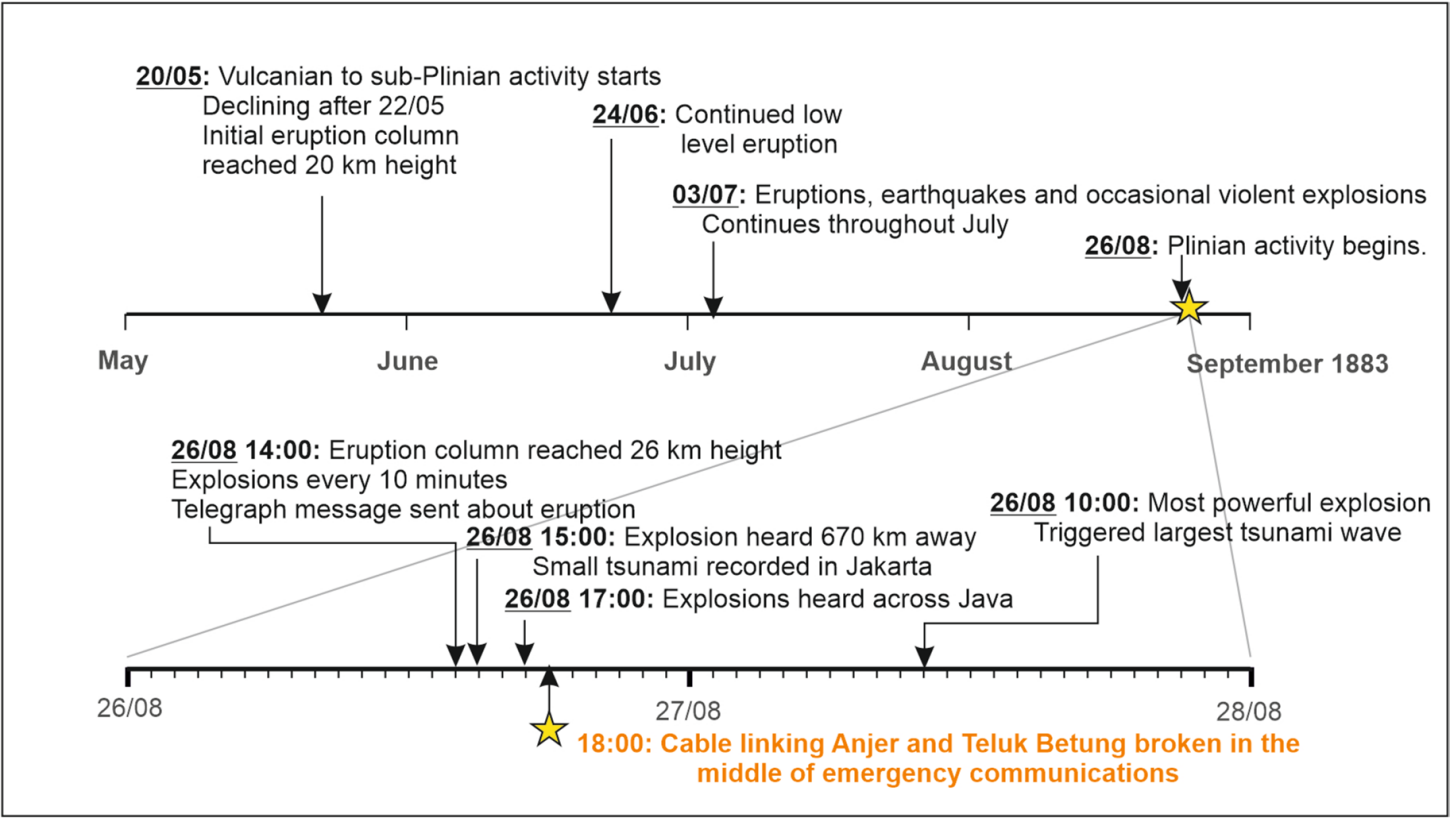
Timeline for the 1883 eruption of Krakatau volcano, Indonesia, highlighting key events in the build-up to the climactic eruption and the timing of damage to the subsea telegraph cable connecting Anjer and Teluk Betung (yellow star) that related to a tsunami, based on accounts and events documented in Verbeek (1885), Latter (1981), Dörries (2003), Madden-Nadeau et al. (2021), and Toivanen (2021)

The VEI 6 eruption of Krakatau volcano (Indonesia) in 1883 was one of the deadliest volcanic eruption episodes in modern history, with a total of 36,000 estimated fatalities (Symons, 1884; Self and Rampino 1981; Self 1992; Deplus et al. 1995; Madden-Nadeau et al. 2021). The extreme destruction and widespread impacts of Krakatau generated broad attention and scientific study, and the episode is notable in that it was the first globally reported volcanic eruption in real-time. Rapid reporting was possible because of a subsea cable connecting Jakarta (then called Batavia) to the global telegraphic network (Fig. 2; Dörries 2003). Shortly after the advent of subsea telegraph cables in the late nineteenth century, a significant expansion of telegraphic connections began in South-east Asia, to connect to administrative centres in Europe (Winchester, 2003). Initially, cables were primarily laid on land in Indonesia; however, the protective insulation material was regularly destroyed by termites, prompting offshore routes to be chosen, which also had the benefit of providing more direct connections (Toivanen 2021). Cables installed in 1859 connected Indonesia to Singapore and then to Malaysia and Australia in 1870, providing the first international communications to the region (Dörries 2003).

The 1883 eruption of Krakatau commenced in late May, with Vulcanian to sub-Plinian activity that created a 20-km-high eruption column on 20 May (Fig. 2; Table S-2; Self 1992). The eruption escalated on 26 August, as reported by a telegraph message that was sent along the subsea cable network from Batavia that reached Europe within a day (Dörries 2003). This was followed by more intense explosions, culminating in tsunamis, during which a cable that linked Anjer and Teluk Betung to the north was cut at 18:00 on 26 August (Fig. 2). Damage was attributed to seafloor entanglement of vessel masts and debris that were sunk during a tsunami. Arrangements were made for repair of the broken cable, which commenced at 01:00 (Toivanen 2021) but were ultimately unsuccessful due to the impacts from the tsunami. As a result of the extreme destruction in Anjer and a lack of capacity to repair the cable station, the cable route crossing the Sunda Strait was abandoned, and a different route selected. Aside from this damaged cable, the wider regional telegraphic network continued to be important through the culminating stages of the eruption. Soon after the paroxysmal 10:00 explosion on 27 August, an overland telegram from Serang (west Java) to Batavia reported the fall of pumice and mud rain, with a message afterwards reporting that Merak, and all telegraph lines west of Serang, had been destroyed. The line from Serang was then interrupted for around 24 h (Simkin and Fiske 1983). The subsea network from Batavia remained active, however, and was important in distributing news globally of the event. Several messages on the late morning of 28 August reported the extreme destruction on the coastlines of the Sunda Strait, including the message at 12:00 that said “where once Mount Krakatau stood, the sea now plays” (Simkin and Fiske 1983).

This event played a role in informing the locations of future cable routes; however, presently, two modern fibre-optic cable systems cross the Sunda Strait in a similar location to that occupied by a telegraph cable in 1883. In December 2018, the eruption of Anak Krakatau (the resurgent ‘child of Krakatau’) resulted in the catastrophic collapse of around half of the island into the ocean, triggering a tsunami with up to 80-m runup height, which caused widespread damage to coastal communities around the region (Hunt et al. 2021). Despite the major tsunami, three other fibre-optic cable systems that lie within 15 km to the south-east of Anak Krakatau and those further away that cross the Sunda Strait all remained intact, which may be due to not only the strongly directional nature of the tsunami, but also as modern telecommunications cables are more robustly constructed with the option of lightweight armour in deep water, and are more carefully installed so as to ensure close coupling with the seafloor. Cables are also now buried, where possible, particularly in shallower waters where effects of tsunamis will be harshest.

#### Mount Pelée, Martinique, May 1902

**Fig. 3:**
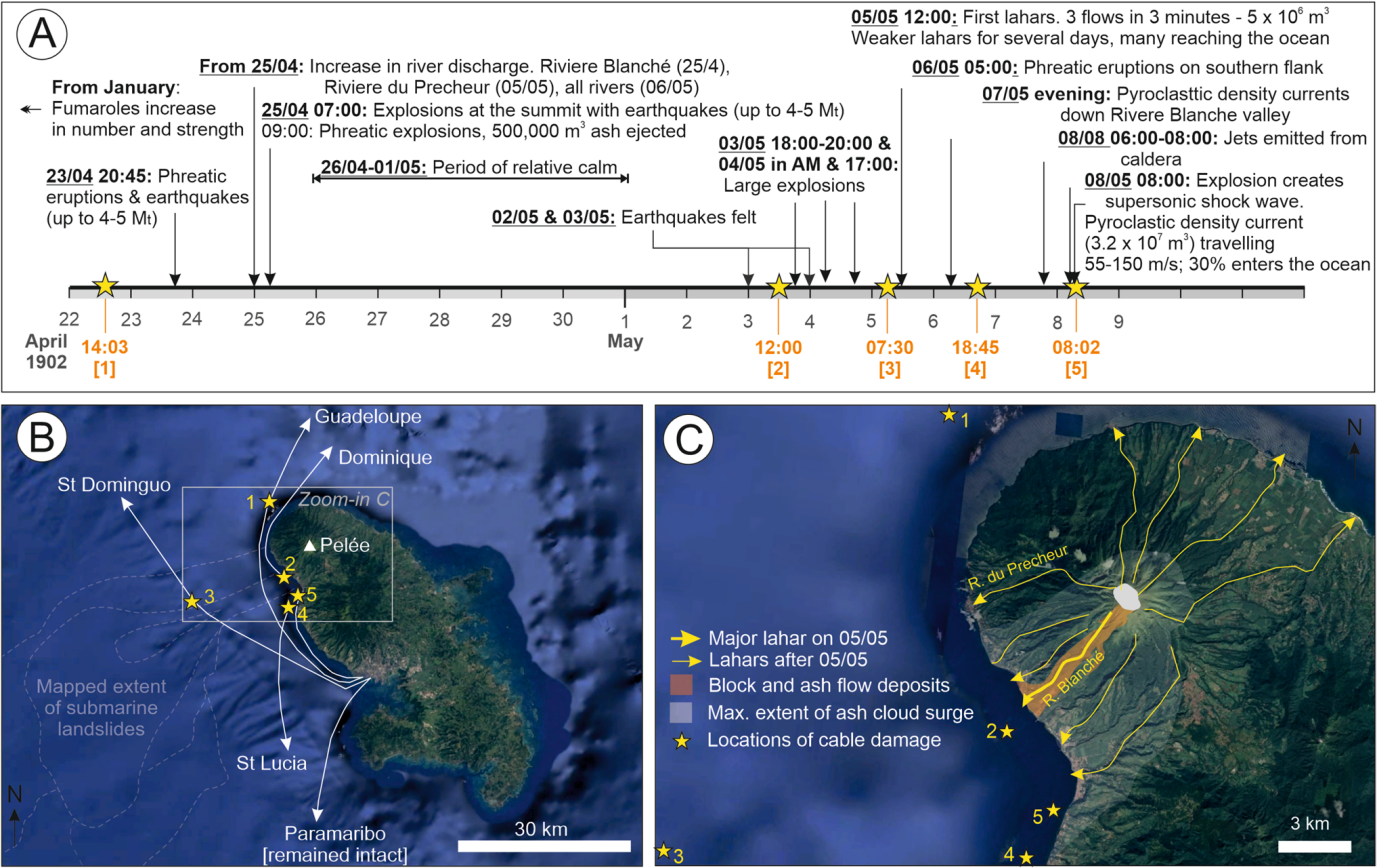
Timeline for the 1902 eruption of Mount Pelèe, Martinique, highlighting** A** the timing of five discrete instances of cable damage (yellow stars) and key events in the build-up to the climactic eruption on the 8 May; **B** the approximate locations of cables (and the locations to which they connected) and extent of older submarine landslides as mapped by Brunet et al. (2016); and** C** detailed view showing the known pathways of lahars and the extent of ‘block and ash flow’ and ‘ash cloud’ deposits that indicate the pathway of the large PDC on 8 May. Timeline based on observations in Westercamp (1987), Chrétien and Brousse (1989), and Tanguy (1994). Background relief based on Google Earth

Mount Pelée, a subaerial volcano on the northern part of Martinique in the Lesser Antilles, began to erupt in 1902, commencing with small phreatic eruptions and up to 4–5 M_w_ earthquakes on 23 April (Fig. 3; Table S-3; Chrétien and Brousse 1989; Lacroix 1903). In the build-up to the eruption climax (8 May; VEI 4), destructive lahars occurred (many of which reached the ocean). A notable lahar on 5 May had an estimated volume of 5 × 10^6^ m^3^, being triggered by either a phreatic eruption or a failure on the south side of the crater lake whose level had risen in the days prior (Chrétien and Brousse 1989). This lahar reached an estimated speed of 33 m/s (120 km/h), having travelled 6 km from the crater to the shore in 3 min, where it temporarily displaced the seawater (Tanguy 1994). The most powerful mass flow was a PDC (3.2 × 10^7^ m^3^; Tanguy 1994; Gueugneau et al. 2020) that is estimated to have travelled up to 155 m/s (558 km/h) and destroyed the city of St Pierre, killing nearly all its inhabitants (Lacroix, 1903; Chrétien and Brousse 1989; Tanguy 1994). The PDC reached the harbour of St Pierre 8 km from the crater (Gueugneau et al. 2020).

At a similar time to the connection of Indonesia, telegraph cable networks were laid across the Caribbean to provide communications with Europe. The earliest subsea cable in the region was laid in 1867 (Hambright 1991). The West India and Panama Telegraph Company installed a network in 1873 that connected Cuba, Jamaica, Puerto Rico, Antigua, Guadeloupe, Dominica, Martinique, St. Lucia, St. Vincent, Barbados, Grenada, Trinidad, and British Guiana (CIAL 2024). Other telegraph companies followed suit, to the point that for many islands there were more telegraph cable connections at that time than there are presently modern fibre-optic cable connections. By the start of the twentieth century, six telegraph cables connected to the volcanic island of Martinique, with landing points at Saint Pierre and Fort de France on the west coast of Martinique to connect key ports and protect them from storms that come in from the east (Fig. 3; Chrétien and Brousse 1989). These cables provided important connections onwards to Dominica, Guadeloupe, Haiti, St. Lucia, and Suriname. Five of these six subsea telegraph cables that connected to Martinique were damaged during the 1902 eruption (Fig. 3; Chrétien and Brousse 1989). The timing of the cable breaks did not correspond to a felt earthquake nor a tsunami (Chrétien and Brousse 1989). While one cable broke 2 min after the most vigorous explosion, most cable damage was asynchronous with the eruption climax. It is thought that the first break, on 22 April, related to submarine slope failure triggered by the initial seismicity and phreatic activity, and hence involved collapse of previously accumulated sediments. No cable damage occurred in the period of relative quiescence, with the next instances occurring between 3 and 6 May, when lahars flowed into the ocean. As the eruption escalated, community leaders were shocked to see two French ships repairing the telegraph cables close to shore, rather than offering to evacuate the island, questioning whether the governor valued telegrams more than human lives (Zebrowski 2002).

#### La Soufrière, St Vincent, May 1902 and April 2021

La Soufrière is a subaerial volcano that lies on the north-west part of St Vincent in the Caribbean. The 1902 eruption of La Soufrière volcano initiated as a period of unusually elevated seismicity between February and April, which prompted initial evacuations from Morne Ronde, the location of at least two cable landings (Table S-4; Anderson and Flett 1903; Cox 2004). Conditions remained similar until lake level changes and phreatic activity were noted at La Soufrière volcano on 5 May (Anderson and Flett 1903; Flett et al. 1908; Pyle et al. 2018), with the eruption climaxing (VEI 4) on 7 May when a powerful PDC was generated and which entered the ocean on the north-western side of the island (Roobol and Smith 1975).

**Fig. 4:**
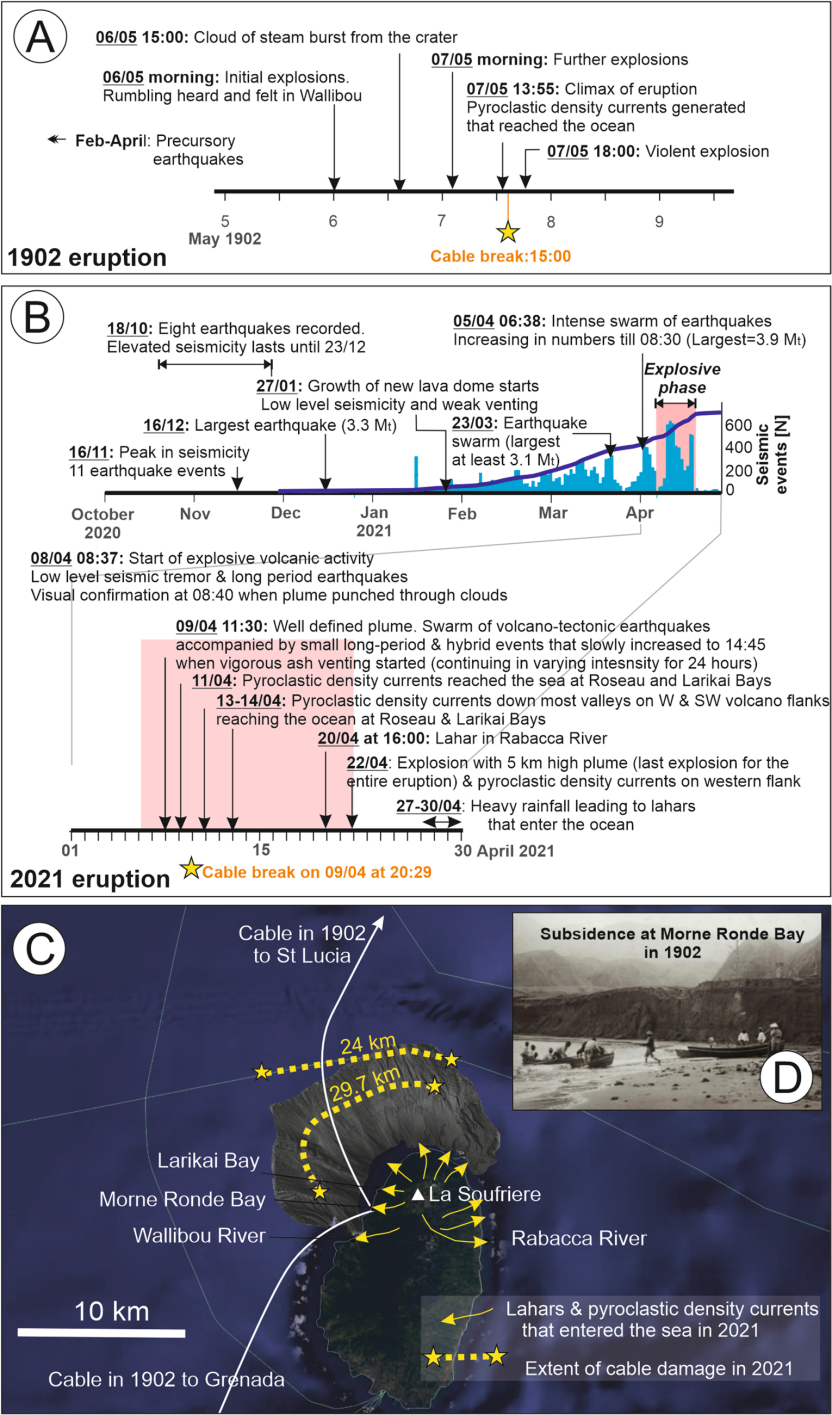
Timeline for eruptions of La Soufrière, St Vincent, and known locations of cable damage, including** A** 1902 eruption during which six telegraph cables were damaged (although the precise timing is only known for one, and the locations of cables remains unclear based on existing information); **B** 2020/2021 eruption during which two cables were damaged (the timing is only known for the cable closest to shore), which includes information on seismicity derived from land-based seismic monitoring; **C** location of cables and extent/site of damage (where known), and pathways of lahars and PDCs that reached the sea during the 2021 eruption phase; and** D** photograph illustrating collapse of shoreline along a 200-m stretch of coastline at Morne Ronde Bay (from Nottingham Evening, 1902). Timeline and locations of events based on accounts and Ronde observations in Foster Huggins (1902), Anderson and Flett (1903), Roobot and Smith (1975), Pyle et al. (2018), Phillips et al. (2024) and Robertson et al. (2024). Multibeam backscatter data to the north of St Vincent that illustrate seafloor relief is from Irvine and Lipsham (2019)

In 1902, St. Vincent was connected by six subsea telegraph cables. The precise location of four of those cables is not well documented in available records, but similarly to nearby Martinique, all cables are known to have reached landfall on the western side of the island (Fig. 4). These cables were all damaged during the 1902 eruption. The telegraph cable that connected on to St. Lucia was damaged during the eruption climax on 7 May, which, according to eyewitness accounts, broke ~ 5 min after the inception of the powerful ocean-entering PDC (Roobol and Smith 1975). However, the other five cables were damaged at some point (precise timings not known) between 5 and 7 May, which includes periods prior to the eruption climax. Their cause is unclear as records are less well developed for this eruption compared to Mount Pelée. Anderson and Flett (1903) reported that a section of the coastline collapsed into the ocean, with the area affected extending 2 km northwards from the mouth of the Wallibou River towards Morne Ronde Bay where pyroclastic sediments had recently accumulated. Soundings performed by the Halifax and Bermudas Cable Company and Direct West India Cable Company revealed that the coastline had retreated by > 180 m, with reports that “boats now travel over the site of Wallibo[u] Village (Scarlett, 2021). At 50 feet [15.2 m] outwards from the present beach, and where land formerly existed 20 feet [6.1 m] above sea level, the water is now 7 ½ fathoms [13.7 m] deep, and at 100 feet [30.4 m] outward from the same point on the beach it is 18 fathoms [32.9 m]. This subsidence appears to be strictly defined with its southern limit at the mouth of the Wallibo[u] River” (Foster Huggins 1902; Fig. 4D). Photographs taken a few months afterwards reveal steep scarps at the landward limit of this evacuated material, which cut into pyroclastic deposits. Soundings made by a cable repair ship revealed that the seafloor had also changed in deeper water, finding that the seafloor had “sunk 1,200 m where it was only 300 m down” (Nottingham Evening, 1902). It is possible that the sudden emplacement of pyroclastic material at the coastline and on the steep submerged slope (average 9°) caused slope collapse. Gravity cores taken from the deep-sea Grenada Basin, up to 100 km to the west of St. Vincent, recovered deposits of pyroclastic material that are geochemically linked with the 1902 La Soufrière eruption (Carey and Sigurdsson, 1978). These deposits are indicative of transport by a turbidity current. It is therefore possible that smaller PDCs and/or lahars that delivered material to the coastline and into the ocean earlier in the eruption sequence primed submerged slope failures and a longer runout turbidity current.

More recently, two fibre-optic cables were damaged following the 2020/21 eruption of La Soufrière, which was also a VEI 4 event (Fig. 4B, C; Robertson et al. 2024). As St Vincent now has five international cables that connect to the south (i.e. far away from the volcano), data traffic could be rerouted through other cables with no discernible impact on telecommunications at the time. During the 2021 eruption, two PDCs and more widespread lahars reached and entered the ocean along river catchments that flow to the west, north, and east (Phillips et al. 2024). The closest cable to shore was damaged on 9 April at 20:29, during a period of voluminous and energetic ash venting accompanied by explosions that peaked between 20:00 and midnight. The precise timing of the more distal cable is not presently known. The total damaged or buried length of the two cables totals 53.7 km and likely relates to the effects of large individual or numerous smaller submarine mass movements. Reports from the cable repair company indicate elevation changes, with several metres of new sediment locally accumulated on a subsea cable that lies around 8 km from shore and with observations of significant accumulations of fresh tree debris around the cable location. Pre-eruption bathymetry indicates a series of linear gullies that show a close alignment with river outflows, indicating that there is a continuation of sediment transport pathways carried by lahars along river catchments on land and extending further offshore. Background relief based on Google Earth

#### Kick ‘em Jenny, July 2015

Kick ‘em Jenny is located around 8 km north of Grenada and is the only reported submarine volcano in the Lesser Antilles arc to be volcanically active in historic times (Devine and Sigurdsson 1995; Allen et al. 2018). It has been responsible for 15 eruptions since its discovery in 1939 (Global Volcanism Program, 2024). Eruption styles have ranged from effusive (dome-forming) eruptions to explosive eruptions (Devine and Sigurdsson 1995). Its proximity and potential risk to coastal populations has meant that the submarine edifice has been surveyed several times since the first in 1962 (Robson and Tomblin 1966). A high-resolution bathymetric survey in March 2002 showed the summit of an active cone 300-m wide with a crater 264-m deep in water depths of around 185 m (Lindsay et al. 2005). The Kick ‘em Jenny edifice is located in a larger horseshoe-shaped structure, which has been attributed to one or more flank collapses (Dondin et al. 2012). The cumulative volume of past flank collapses is estimated at 10 ± 0.5 km^3^ (Allen et al. 2018).

**Fig. 5:**
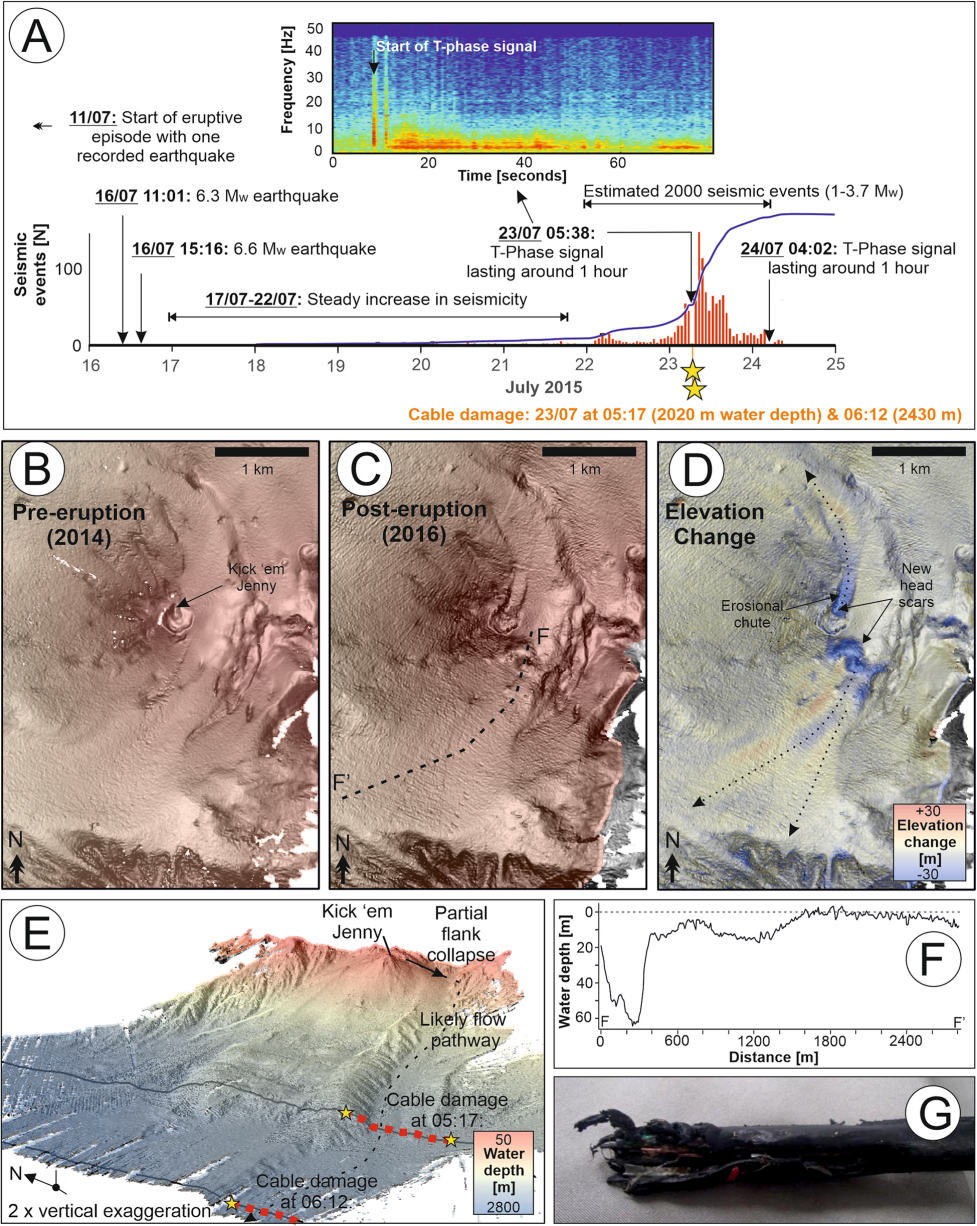
Timeline and context for 2015 eruption of Kick ‘em Jenny and associated damage to two subsea cables. Observational time series data (**A**) are based primarily on information derived from land-based seismic monitoring network, which includes an hour-long T-phase signal (inset shows focus on the first minute of that signal from Latchman et al. 2023) that coincides with when the cable damage occurred. Maps of seafloor relief illustrate the seafloor morphology before (**B**), in 2014, and after (**C**), in 2016.** D** Change in elevation from 2014 to 2016. Two collapse scars on the volcano flank are evident: one on the south-east flank that produced a mass flow that flowed to the south-west where it reached a submarine channel as shown in the 3D seafloor rendering in **E** where the inferred pathway of the flow that damaged the two seafloor cables is annotated. A profile of seafloor elevation change between 2014 and 2016 along the collapse on the south-east flank is shown in** F**. A photograph of the northern-most recovered end of the cable closest to Kick ‘em Jenny is shown in** G**. Seafloor surveys are from Allen et al. (2018)

The University of West Indies Seismic Research Centre monitors the activity of Kick ‘em Jenny using a network of land-based seismometers (Dondin et al. 2019). A notable unrest episode commenced on 11 July 2015, with > 6 M_w_ earthquakes recorded on 16 July, and a steady increase in seismicity until 23 July when the eruption reached its climax over the course of an hour at around 06:00 (Fig. 5A; Latchman et al. 2023). Two subsea fibre-optic cables were damaged on the same day (23 July), adversely impacting internet connections between Grenada, St. Vincent, Barbados, and Trinidad (Fig. 5A, E). Trinidad had one unaffected subsea cable, which provided critical back-up, demonstrating the importance of geographic diversity in cable routing. The cable damage was recorded first with a cut at 2020-m (at 05:17) and then 2430-m water depth (at 06:12), at straight-line distances of 12 km and 18 km respectively to the west of Kick ‘em Jenny (Fig. 5E, G). Damage affected a 3–4-km length of each cable, as noted during the repair operations. Repairs were initially delayed until it was deemed safe to enter an exclusion zone around the volcano and completed on 4 August (TSTT 2015; TechNews, 2015). The timing of both instances of cable damage precedes the peak in seismicity that represents the climax of volcanic unrest (Fig. 5A; Latchman et al. 2023).

An hour-long T-phase signal was recorded at land-based seismometer stations starting at around 05:38–05:44 (Fig. 5A; Allen et al. 2018; Latchman et al. 2023). While T-phase signals have been linked to submerged volcanic explosions, the unusually long duration and the fact that this T-phase signal progressively increased in intensity over the first 10 min indicate that this signal may instead have been caused by a submarine mass movement (Allen et al. 2018; Latchman et al. 2023). Seafloor surveys performed in 2014 and 2016 reveal debris flows occurred on the southern flank of the volcano, as a result of partial flank collapse. These events emplaced remobilised material over an area of 9.6 × 10^3^ m^2^ with an average thickness of 10 m towards the south-east (as determined by Allen et al. 2018; Fig. 5B–F). This collapse was likely primed by oversteepening of the flank of Kick ‘em Jenny during an earlier eruption in 1988, and ultimately triggered by intense seismic activity in 2015 (Latchman et al. 2023).

These observations appear to confirm the prior hypothesis that the hour-long T-phase signal was caused by slope collapse and/or the resultant runout (and not a volcanic eruption itself), particularly because it is bounded by the timings of the two cable breaks. The deep-water cable damage occurred within a submarine channel that connects to the upslope area of this mapped landslide (Fig. 5E). The delay of 55 min between the two cable breaks that occurred 6 km apart reveals a speed of around 6.5 km/h (or 1.8 m/s), which is surprisingly slow compared to speeds of other cable-damaging flows elsewhere (e.g. Carter et al. 2014; Talling et al. 2022; Clare et al. 2023a). To explain the timing, rather than a single large collapse, this could have been a series of smaller landslides which built up debris at minor slope breaks before triggering further cascades, or a series of collapses with initial ones depositing with lower runouts and later flows travelling successively further on the surfaces smoothed by earlier deposits. A likely flow pathway along the submarine channel in which the cable faults occurred indicates a runout distance of at least 18.9 km, exceeding the previously reported 1.5 km (Latchman et al. 2023). Examination of the deepest water cable at its northernmost break location revealed abrasion of the outer insulation as well as a cut (Fig. 5G); hence, this damage may have been caused by a combination of snagging of the cable on the irregular and rocky channel walls and dragging by a sediment density flow.

#### Soufrière Hills dome collapse, Montserrat, 1997

The Soufrière Hills volcano lies on the southern part of the island of Montserrat, which is at the northern end of the Lesser Antilles. Since 1995, the Soufrière Hills volcano has been in a semi-constant eruptive state, with variations in eruptive output as represented in a series of dome growth and collapse phases (Aspinall et al. 1998; Jackson et al. 1998; Young et al. 1998; Table S-5). Over 65% of the pyroclastic material that was produced during this eruption so far has entered the ocean (Trofimovs et al. 2006). A major dome collapse (8.5 × 10^7^ m^3^) occurred in late September 1997 during a period of enhanced explosive activity, with an even larger collapse in 2003— the largest historically recorded, worldwide, and transported > 2.1 × 10^8^ m^3^ of material as a PDC that reached the coast (Trofimovs et al. 2006). Where this latter flow reached the ocean, it separated to trigger a PDC over the sea surface and a turbidity current that travelled at least 20 km along the seafloor (Herd et al. 2005; Edmonds and Herd 2005; Trofimovs et al. 2006).

Prior to 1995, Montserrat was connected by the undersea branch of the Eastern Caribbean Fibre System. However, the cable was purposefully cut in 1997 when the southern part of the island was evacuated and the cable landing station in Plymouth was subsequently destroyed by PDCs. In the absence of a fibre-optic cable connection, Montserrat had to rely on an expensive and precarious microwave link with Antigua, which had major impacts on economic growth. It was only in October 2020, 25 years after the initial disconnection, that a new fibre-optic cable was installed to restore digital communications and access to high-speed broadband (Digwatch 2020). This new cable landfalls on the north-west coast of the island and is routed offshore to the north of the island as a branch off the Eastern Caribbean Fibre System that connects Montserrat, to Antigua to the north, and Barbuda and Guadeloupe to the south (Government of Montserrat, 2020). This provides greater resilience, by providing diverging connections to other countries and avoids offshore areas that could be affected by resurgence of the active volcanic centre on Montserrat.

#### Esporão do Monaco Submarine Volcanic System, Azores, in 1902 and 1907

The Azores archipelago lies in the North Atlantic Ocean and comprises nine volcanic islands (Madureira et al. 2005). At least 28 historical Hawaiian to sub-Plinian volcanic eruptions have been recorded in the Azores, of which 15 are located on land (Gaspar et al. 2015). Evidence of submarine eruptions is mostly based on short-lived islet-building Surtseyan eruptions, as witnessed in 1638 and 1811 on São Miguel Island, 1720 on Dom João de Castro Bank, and a significant event in 1957–1958 at Faial Island that began as a Surtseyan eruption but which progressed to Strombolian when the vent extended above sea level (Machado 1959; Cole et al. 2001; Gaspar et al. 2015). Evidence for other submarine eruptions has come from two instances of subsea cable damage; however, the absence of any subsea monitoring means that any information on the nature of these eruptions is not known.

**Fig. 6:**
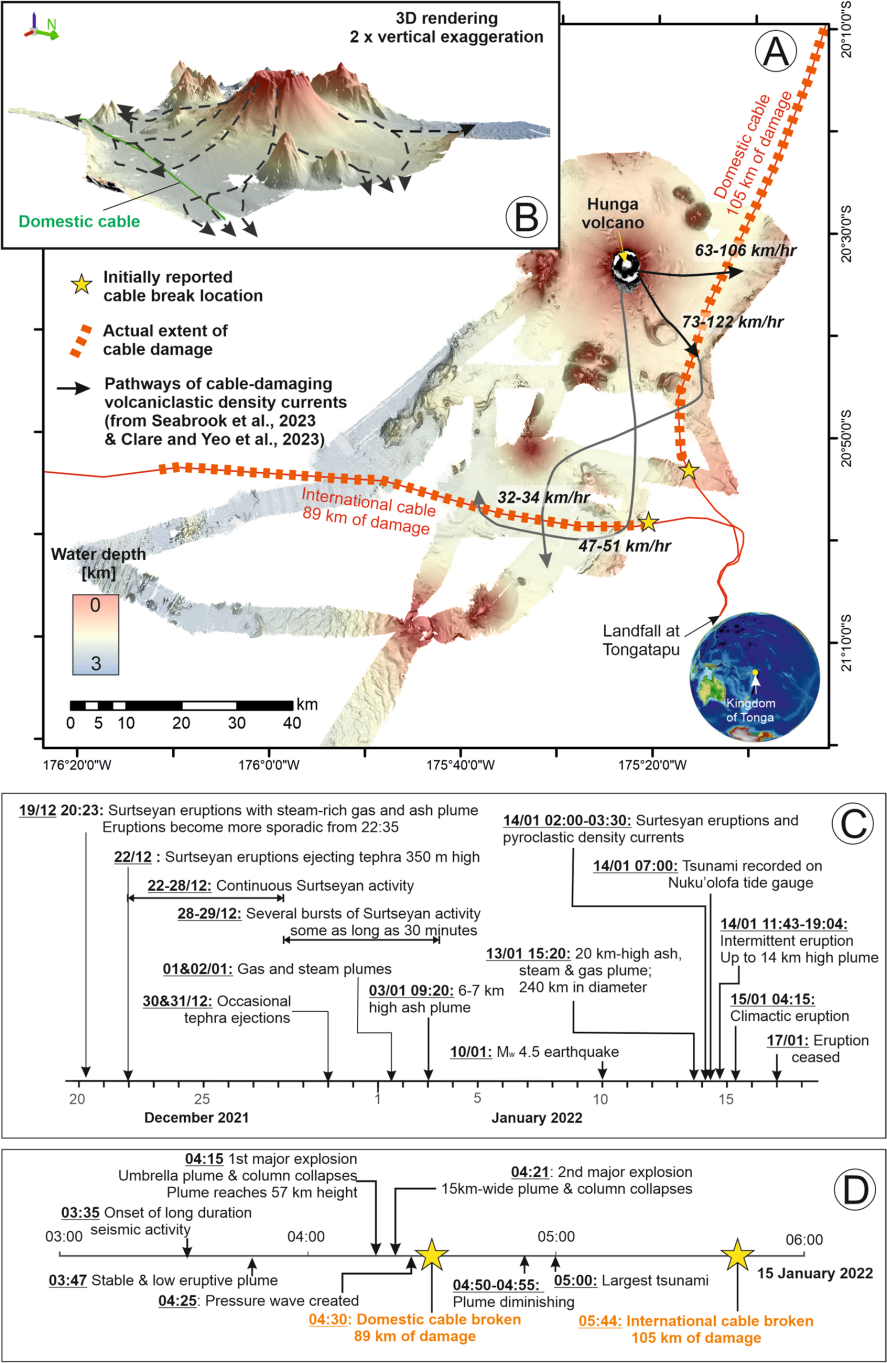
Timeline and context for 2021–2022 eruption of Hunga volcano, Kingdom of Tonga.** A** Location and extent of subsea telecommunications damage and/or burial and indicative pathways of volcaniclastic density currents that intersected with cables overlain on bathymetric data acquired 3 months after the eruption.** B** Three-dimensional rendering of Hunga volcano illustrating the many pathways likely taken by volcaniclastic density currents that radiated from Hunga volcano during the eruption on 15 January 2022, which were steered by pre-existing relief (based on Seabrook et al. 2023 and Clare et al. 2023a). **C** Timeline of the eruption of Hunga volcano from inception in December 2021, to completion on 17 January 2022, and detail **D** on the climactic phase of the eruption when the two subsea cables were damaged

A submarine telegraph cable that connected the Terceira and Pico islands to the south-west of Terceira was damaged between 7 and 8 May 1902. The cable repair operation identified multiple ruptures in the subsea cable that occurred along a section of about 16-km length between water depths of 450 and 1400 m (Chaves 1915; Weston 1964). On 1 April 1907, a cable that connected São Miguel and Faial islands was cut at a water depth of 400 m to the south-west of São Miguel (Fig. 6; Gaspar et al. 2015). Seafloor sediments sampled from areas of buried cable were identified as ‘fine dust pumice’, with observations of anomalously high temperatures at the seafloor (Chaves 1915; Weston 1964). In these two cases, the precise location of the volcano relative to the cables is not accurately known, and with no information on the eruption itself, the determination of what aspect of the eruption may have led to cable damage remains purely speculative.

#### Mihara-yama volcano, O-shima-jima (1986), and Oyama volcano, Miyake-jima, Japan (2000)

Several of the approximately 300 inhabited islands across the Japanese archipelago are active volcanic islands (Utada et al. 2007; Maeno et al., 2022). These include O-shima and Miyake-jima, which are part of the 300-km-long Izu Island chain that lies along the Izu-Bonin-Mariana Arc, south-east of mainland Japan. Two volcanic eruptions along this chain are relevant to this study. The first is on Miyake-jima island, at the centre of which lies Oyama volcano, which has erupted multiple times historically, including notable eruptions in 1940, 1962, and 1983 (Arai 2018). After 17 years of quiescence, Oyama volcano started erupting on 27 June 2000, with a large phreatomagmatic explosion on 18 August. From 29 August, gas venting occurred from the centre of the volcano, and several PDCs ran down its flanks (Arai 2018).

The second relevant eruption is of Mihara-yama volcano, which lies at the centre of O-shima. Mihara-yama has erupted multiple times historically, including small to medium eruptions in 1950, 1957, and 1974, with the largest eruption in 1778 (VEI 4; Kozono et al. 2022). On 15 November 1986, a large-scale eruption commenced, with frequent explosions on 21 November, with lava flows encroaching close to settlements (Kozono et al. 2022). Eruptions became more frequent and vigorous across the island; hence, an evacuation order was issued for all 10,500 island residents by 23:00 on 21 November. This was the largest scale evacuation to date in Japan (Arai 2018).

Significant investment in infrastructure has led to a network of telecommunications cables that connect relatively remote islands across Japan (Arai 2018). The first subsea telegraph cable was laid between O-shima and Shimoda in 1903, and a subsequent connection was installed to Tokyo in 1904. In 1906, cables were laid to connect O-shima to Miyake-jima and across the wider Izyu islands and upgraded in 1934 to a telephonic network (Nippon Denshin Denwa Kosha, 1971). By the early 2000 s, a fibre-optic cable system provided the main mechanism for telecommunications for most islands, but with an ancillary microwave radio channel for some of the islands not served by a fibre-optic cable.

In the case of the 1986 eruption of Mihara-yama volcano, the entirety of the 3700 island residents were evacuated by 4 September, and remained off the island until February 2005 (Arai, 2018). While all staff from the local government, Tokyo Electric Power Company, and NTT-East (the telecommunications company) were also evacuated, these key support personnel remained near the island aboard a passenger ship to ensure communications services kept running until 26 September when volcanic gas emissions became too hazardous. At this point, the commercial power system for the island could no longer be maintained, leading to an outage that continued until November 2004 (Arai 2018). Telecommunications via the subsea cable were initially kept going after the commercial power outage by emergency generators run by NTT-East; however, these could not be maintained indefinitely. The telecommunications system in Miyake-jima stopped working on 27 December 2000 (Arai, 2018). As the fibre-optic cable also supported microwave communications, this also cut off the surrounding islands that relied upon them (Arai, 2018). To address this issue, two new fibre-optic cables were installed to connect Miyake-jima, which could be powered from their other landing points on mainland Japan and the nearby island of Hachijo-shima, without a need for maintenance on Miyake-jima. Additional emergency portable microwave radio and satellite devices were installed on nearby islands to ensure basic telecommunications services to all islands (Arai, 2018). A further subsea cable was installed in 2008 to provide direct connection to the islands previously reliant solely on microwave communications.

In contrast, the implications of the Oyama volcano eruption in 2000 were less severe. Three employees of the Tokyo Electric Power Company remained on the island to ensure power supply to the island was maintained while residents were evacuated, which enabled telecommunications traffic to continue. Telephone traffic reached five to seven times its normal levels during the climax of the eruption, but with no failures, enabling essential seismic monitoring to continue (Arai 2018). Without the three power company employees on the island, the network would have become disconnected, as was the case in Montserrat in 1997. In both of these instances, there was no physical damage to the telecommunications infrastructure. Instead, the vulnerability was due to reliance on the onshore power supply.

#### Hunga Volcano, Kingdom of Tonga (2022)

The Hunga Volcano lies in the waters of the Kingdom of Tonga, and is one of the hundreds of volcanoes that occur along the Tonga–Tofua–Kermadec Arc in the South Pacific Ocean (Seabrook et al. 2023). The two small islands of Hunga-Tonga and Hunga-Ha’apai presently form the subaerial expression of the approximately 2-km-tall edifice, which features a 5-km-wide caldera at its summit (Le Mével et al. 2023). Surtseyan eruptive episodes were documented in 2009 and 2014–2015, with activity focused within the caldera itself (Colombier et al. 2018; Garvin et al. 2018; Brenna et al. 2022; Le Mével et al. 2023). The eruptive episode in 2015 formed a 120-m-high and 2-km-wide tephra cone that joined the two islands together (Garvin et al. 2018). After a period of quiescence, a more recent and vigorous eruptive episode commenced as explosive eruptions on 19 December 2021, and which suddenly escalated on 15 January 2020 as a VEI 6 eruption with a 57-km-tall eruption plume (Fig. 6; Table S-6; Lynett et al. 2022; Millan et al. 2022; Wright et al. 2022 Borrero et al. 2023).

Two subsea telecommunications cables connect to the island of Tongatapu in Tonga: one domestic cable linking to the island groups to the north, and an international cable, connecting to Fiji and onward to Australia, providing the only link to the wider global network. Both cables were broken on 15 January, with the domestic cable severed at 04:30 and the international cable at 05:44 (Fig. 6D; Clare et al. 2023a). Repeat seafloor surveys before and after the eruption revealed that a volume of > 6 km^3^ (likely around 8 km^3^) was evacuated during the eruption and provide evidence of erosion (up to 100 m deep) focused within steep linear gullies that radiate from the caldera (Fig. 6A, B; Seabrook et al. 2023). This intense, focused erosion involved the excavation of a further 3.5 km^3^ of seafloor sediments within 10 km of the caldera (Seabrook et al. 2023). Widespread deposition of volcaniclastic material blanketed the seafloor in deeper water. The cable damage is interpreted to have been caused by volcaniclastic density currents that were triggered by the rapid delivery of large volumes of pyroclastic material directly into the ocean as the lower part of the eruption column collapsed (Clare et al. 2023a). Volcaniclastic density currents were steered along pre-existing relief, and first damaged the domestic cable within a valley to the east of the volcano, and then around an hour later reached the international cable. The timings and locations of cable breaks indicate maximum speeds of between 17.6 and 33.8 m/s (Clare et al. 2023a). Seafloor sampling revealed that flows reached hundreds of kilometres from the caldera (Seabrook et al. 2023; Beinart et al. 2024). A length totalling 194 km of telecommunications cable was damaged and/or buried by these far-travelling flows (Fig. 6A). When backup satellite links kicked in 5 days later, data capacity was < 1% of normal levels. It took 5 weeks to repair the international cable due to the remoteness of the damage, and 18 months for repair of the domestic cable to restore internet connections to island groups north of the main island of Tongatapu.

As part of routine hazard assessments for new systems, the subsea cable industry attempts to avoid routing close to volcanoes wherever possible. However, in volcanic settings such as at Hunga volcano and the other case studies we have discussed, avoidance is simply not possible and may not be done as the likelihood of such an event is relatively rare— particularly compared to the typical 25-year design life of a subsea cable. Prior to its 2022 eruption, Hunga volcano (and others along the arc) was identified by the cable owner as a potential hazard; however, what was surprising was the sheer extent of damage to subsea cables and the runout distance of the damaging flows, which far exceeded anything that was previously thought. This meant that there was insufficient spare cable in stock for the repairs, and even when three other subsea cable owners provided lengths of cable (89 km total) for the repair of the international cable, there was insufficient cable to fix the domestic system, which required procurement of a brand-new cable. The 18-month delay arose from repair timescales comprising 7 months for manufacture of the replacement cable length, 4 months for shipping, 2 months for the cable ship to get to Tonga, and 8 days to physically perform the repair. These combined factors have prompted the cable owner and the wider subsea cable industry to recognise that such hazards can be far wider-reaching than previously thought and prompted a need for greater investment in both remedial measures (e.g. more spare supplies) as well as in back-up communications systems.

## Discussion

We now synthesise the different case studies to provide a broader understanding of the processes responsible for damage, and which other volcanoes could pose a threat to subsea cables. We then summarise some of the outstanding challenges and uncertainties and discuss some of the strategies to address them, in relation to enhancing network resilience and improving our broader understanding of volcanic hazards, particularly for remote islands.

### Multiple different processes can damage cables during volcanic eruptions

**Fig. 7:**
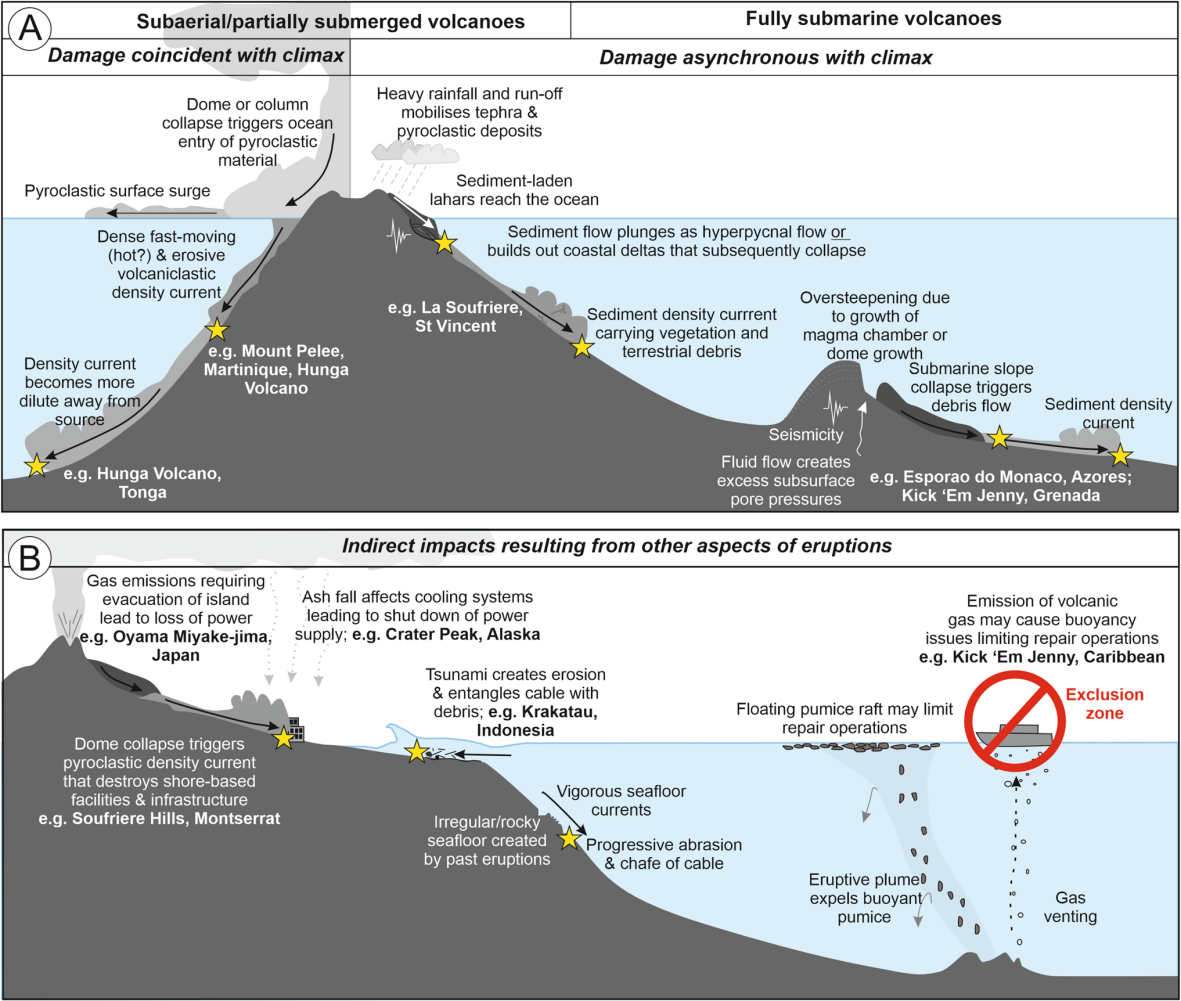
Schematic diagram showing different aspects of volcanic eruptions and associated hazards that can damage subsea cables. **A** Direct impacts associated with volcanic eruptions, including those that coincide with an eruption climax and those that occur asynchronously. **B** Indirect effects of volcanic eruptions that can impact subsea telecommunications connections

The damage-causing processes for submarine cables are distinct from those that damage terrestrial telecommunications infrastructure, which are primarily related to ash fall. During most eruptions that occur over days–weeks, several different hazardous processes can occur in sequence or in parallel, as attested by multiple cable breaks attributed to different processes during the eruption of La Soufrière , 1902. The case studies in the section “Results” reveal that all the observed volcanic eruptions that resulted in damage to subsea cables involved episodes of extreme rates or volumes of volcanic sediment and/or rock transport into and within the ocean, with most examples originating from volcanic islands (Fig. 7). Our analysis reveals that mass flows generating submarine cable breaks include those resulting directly from subaerial processes, those that transition from subaerial to submarine, and those that are solely submarine in origin, including:

(i) Long runout (sometimes > 100 km) submarine volcaniclastic density currents triggered by submarine fountaining and eruption column collapse into the ocean, which can drag, abrade, or excessively bury cables over extreme lengths (e.g. offshore Hunga Volcano, Tonga).
(ii) Land-sourced PDCs from dome-collapse or related Vulcanian eruptions that can destroy shore-based cable landing stations (e.g. Soufrière Hills, Montserrat in 1997) or enter the ocean to initiate submarine volcaniclastic density currents (e.g. Mount Pelée, 1902). Other mechanisms that can trigger PDCs include caldera collapse, phreatic explosions, pyroclastic fountaining, lateral blasts, and partial or total eruption column collapses onto land.
(iii) Lahars that enter the ocean may directly transform into submarine sediment density currents where they plunge and flow along the seafloor due to their high sediment concentration. Successive laharic sedimentation may also rapidly build up unstable progradational deltas at the shoreline, which readily subsequently collapse to generate submarine density currents (e.g. La Soufrière, 1902). Such lahars may occur during the eruption or occur many months or years after the main eruption due to eruption-induced changes in the volcanic landscape and drainage patterns and triggered by heavy rainfall (Massey et al. 2010; Phillips et al. 2024).
(iv) Submarine slope collapse on a volcanic edifice or adjacent slopes arising from seismicity, deposition of volcaniclastic debris, changes in seafloor slope resulting from pressurisation, or growth in the magma chamber (e.g. Kick ‘em Jenny in 2015).
(v) In addition to emplacing material, sediment flows triggered by the above processes can be highly erosional, creating large scours, excavating channels, or driving the migration of bedforms. This is particularly the case on steep proximal slopes, where breaks in slope or narrowing of topographic confinement occur, or where flows accelerate (e.g. Crutchley et al. 2013; Karstens et al. 2019; Kuhn et al., 2024). Such erosion may undermine seafloor cables, leaving them unsupported in ‘free span’, vulnerable to impacts by seafloor currents and other processes.
(vi) Impacts resulting from the passage of a tsunami due to abrasion, drag, or entanglement with debris (e.g. Krakatau, 1883). A tsunami may be triggered by a number of mechanisms during an eruption, including seawater displacement by flank or sector collapse, ocean-entry of a PDC, or a pressure wave created by an explosive eruption.
(vii) Impacts on terrestrial infrastructure can also cascade to affect data traffic on subsea telecommunications cables, such as where an island is evacuated or if the power grid shuts down (e.g. Miyake-jima, Japan, 2000).

### What actually causes a cable break?

Beyond determining the timing of a cable break relative to a volcanic hazard, we now discuss the mechanics that may create the damage itself. Damage to a cable may result from a number of factors that are not necessarily unique to the different hazards discussed in the section “Multiple different processes can damage cables during volcanic eruptions”. A density current may expose a cable to a drag force that exerts excessive strain on the cable, which is a function of the velocity and the concentration of the current and the surface area of the cable exposed. This may explain some of the damage; however, it is unlikely to be the only cause. In many of the case studies, the drag force imparted by sediment density currents would undoubtedly be high, yet damage likely also relates to abrasion from coarse material carried by a flow rubbing against the cable, direct impacts by even larger material (e.g. cobbles or boulders), the potential effects of heat (in the case of PDCs), as well as snagging of a cable on volcanic rock exposed as seafloor, and excess burial by rapid sedimentation that can put undue strain on a cable. Other instances of cable damage in non-volcanic settings reveal fairly consistent evidence of cable damage by sediment density currents that exceed a threshold of 5 m/s (e.g. in the Congo Canyon (West Africa) and Gaoping Canyon (Taiwan) (Talling et al. 2022). However, these locations are not perfect analogues as they involve different triggers, flows that were initially cold rather than hot, and distinctly different types of sediment. In one of our highlighted cases (Kick ‘em Jenny), the density current speed may have been much lower than this threshold, with evidence of abrasion of the cable on a steep rocky channel wall, which indicates that snagging of the cable was likely fully or partly attributable to the damage. Therefore, the precise mechanics that cause damage may be complex and it is challenging to determine which combination of these may be responsible in each of the case studies discussed.

### Cable breaks tend to occur around the climax but may happen at any stage of an eruptive episode

Subsea cable damage often, but not always, coincides with the peak of an eruption climax, and may also occur at several different times within an eruptive episode. In the case of the 2022 Hunga volcano, cable breaks occurred very soon after (15–89 min) the peak in mass eruption rate of the eruption, due to volcaniclastic density currents generated when the eruption column collapsed into the ocean. Some instances of damage can also precede an eruptive climax. In the case of Krakatau in 1883, cable damage occurred 16 h prior to the largest explosive eruption, during one of the early phases of tsunami-generating activity. The La Soufrière eruption in 1902 saw damage to cables over a time window of 16 days prior to the main eruptive climax, with cable breaks resulting from a combination of many different processes that initiated at sea (i.e. submarine slope failures) and processes that initiated on land and entered the ocean (i.e. PDCs and lahars). Cable breaks may occur a long time after climactic eruption phases, in the case of changes to volcanic slopes during magma intrusion (before events or without eruptive events), and post-eruptive collapses from sediment fans and deltas.

As revealed by case studies where multiple breaks can occur during a prolonged eruptive episode, the time window over which cable damage can occur is also distinct from many other instantaneous events, such as earthquakes. This unpredictability is problematic as cable faults can occur in the build-up to, and the aftermath of an eruption climax, because of the myriad hazardous phenomena that can occur, such as seismicity, ground deformation and slope instability, ocean entry of fluxes of pyroclastic material, and lahars. With major changes in vegetation and surface hydrology associated with large eruptions, lahars and enhanced fluvial sediment transport processes feed large volumes of mass to the ocean for many years. In this case, even if a cable was repaired following a break that occurred early in an eruption sequence, it may be damaged by subsequent hazards. For example, Montserrat could plausibly have experienced recurrent cable damage over several years, had the system been repaired each time.

### There does not appear to be an explosivity threshold that explains when cable breaks occur, but the extent of damage is greater for larger eruptions

The volcanic eruptions that led to cable damage not only include some of the most explosive instrumentally recorded events (e.g. VEI 6 Krakatau, 1883; VEI 5–6 Hunga volcano, 2022), but also include much less explosive eruptions, with cable breaks sometimes occurring during eruptive activity that does not exceed VEI 0–1. Therefore, there does not appear to be an explosivity threshold for cable-damaging events to occur. A global study of subsea cable breaks similarly concluded that no obvious magnitude threshold exists for earthquake-related damage, and instead found that a combination of ground shaking (which can range from small to large (*M*_w_ 3–9) earthquakes) and sufficient accumulation of sediments on submerged slopes is required to generate cable-damaging mass movements (Pope et al. 2017a). In both earthquake- and volcanic eruption-related cases, the cause of subsea cable damage is primarily attributable to mass movement processes (e.g. sediment density currents or slope failures). Damage is more likely to occur where there is a trigger for such a mass movement (e.g. seismicity or dome collapse) and where there is sufficient sediment available. While there does not appear to be an explosivity threshold for cable damage to occur, the potential footprint of cable damage in our case study examples is generally greater for larger eruptions. For example, the total damaged length of subsea cables from the VEI 5–6 Hunga volcano eruption was 194 km, from the VEI 4 La Soufrière eruption was 57.5 km, and from the VEI 0 Kick ‘em Jenny event was 12 km.

So, why might there be a relationship between the extent of damage and the magnitude of an eruption? Larger eruptions can result in more hazardous mass movement events, particularly as they erupt greater volumes of volcaniclastic material that can trigger large and fast volcaniclastic flows in a number of directions, as a result of dome or eruption column collapse (e.g. Hunga volcano), or from the generation of lahars that remobilise recently deposited ash fall and PDC deposits; and/or (ii) generate greater ground deformation and seismicity that can oversteepen slopes and cause ground motions that can cause submerged slopes to collapse (e.g. Mount Pelée). This combination of factors may provide an explanation for a correlation between eruption size and the extent of damage.

### Not all major explosive volcanic eruptions result in subsea cable damage

In addition to focusing on case studies of documented cable damage during an eruption, it is also important to understand whether eruptions of similar magnitude necessarily lead to cable damage. To determine this, we extracted eruptions of VEI 4 and greater (i.e. large to very large explosivity indices) from GVP (2024), which occurred during a time when a fibre-optic cable is known to have been installed offshore (within an arbitrary 400 km) from the associated volcano (Table 3). We specified an arbitrary 400 km as this captures the documented runout of flows associated with volcanic eruptions in the examples we present in this review and as it provides a conservative upper bound (including far-travelling flows from the Hunga 2022 eruption; Chaknova et al. 2025). It is worth noting that the vast majority of volcanic mass flows tend to have runouts well below this, typically ranging from a few kilometres to tens of kilometres. We can only do this for the fibre-optic cable era and exclude telegraph and coaxial cables as there are insufficient records available to determine the times when those other types of cables were installed and in service. A total of 18 eruptions of > VEI 4 occurred when nearby cables were in service, of which only two had a documented impact (i.e. La Soufrière in 2020 and Hunga volcano in 2022) which were the closest to a cable (12–16 km). Other eruptions that did not result in damage were all located > 42 km away and the eruptions did not result in mass flows entering the ocean (e.g. PDCs, lahars) with most of those flows confined to land or focused away from the location of the cables. The fact that there are relatively few cable breaks related to volcanic eruptions is, to a large part, thanks to the careful routing of subsea cables that takes into account a wide range of natural hazards (including active volcanic areas and features) based on desk-based studies and seafloor surveying. For example, the Caribbean Regional Communications Infrastructure Program (CARCIP) cable in the Caribbean was specifically routed to the west of most of the islands to minimise the impact of tropical storms that come in from the east, but around the area of Kick ‘em Jenny and the nearby Kick ‘em Jack volcanoes (also Grenada), the cable was specifically routed to the east to provide a resilient route (Irvine and Liphsam, 2019). However, avoidance is not always possible, such as where a cable is required to connect to a volcanic island (e.g. Montserrat, St Vincent, Martinique).

**Table 3:** Large and very large (VEI > 4) eruptions that occurred during a time when fibre-optic cables were installed and in service within 400 km of a volcano. It is important to note that VEI is rarely or poorly known for submarine eruptions; hence, these are under-represented in GVP (2024) upon which the VEI values are based

Volcano name	Distance of volcanic centre from nearest shoreline (km)	VEI	Eruption start year	Distance to nearest subsea cable (km)	Fibre-optic cable at the time?	Documented cable damage?
Kasatochi, Alaska	1 km	4	2008	224	Yes	No
Okmok, Alaska	10 km	4	2008	267	Yes	No
Eyjafjallajokull, Iceland	11 km	4	2010	77	Yes	No
Nabro, Eritrea	64 km	4	2011	89	Yes	No
Grimsvotn, Iceland	65 km	4	2011	175	Yes	No
Sinabung, Indonesia	80 km	4	2013 and 2019	86	Yes	No
Semeru, Indonesia	35 km	4	2014 and 2017	66	Yes	No
Manam, Papua New Guinea	5 km	4	2014	68	Yes	No
Kelud, Indonesia	40 km	4	2014	138	Yes	No
Calbuco, Chile	27 km	4	2015	125	Yes	No
Wolf, Ecuador	8 km	4	2015	353	Yes	No
Ulawun, Papua New Guinea	10 km	4	2019	71	Yes	No
La Soufrière, St Vincent	3 km	4	2020	12	Yes	Yes
Taal, Philippines	15 km	4	2020	42	Yes	No
Fukutoku-Oka-no-Ba, Japan	Submerged	4	2021	78	Yes	No
Hunga, Tonga	Submerged	5	2021	12	Yes	Yes

### New scientific insights into the submarine aspects of volcanic eruptions gained from subsea cable breaks

Our understanding of the often-dynamic seafloor environments around coastal volcanoes, volcanic islands, and fully submerged volcanoes is relatively poor, due to limited coverage of detailed seafloor surveys and offshore monitoring in many of the regions where they occur (Goff and Terry 2016). This limitation is particularly acute in the South Pacific Ocean, which explains why the sudden escalation of the Hunga volcano eruption in January 2022 came as a relative surprise (Terry et al. 2022). Repeat multibeam bathymetric surveys that document the seafloor impacts of volcanic eruptions exist in only a few places worldwide and provide insights into the behaviour and extent of volcaniclastic density currents (e.g. Montserrat, Trofimovs et al., 2006; Hunga volcano, Seabrook et al. 2023; and Stromboli, Italy, Casalbore et al. 2022) and of slope collapses of submerged volcanic flanks (e.g. Kick ‘em Jenny, Allen et al. 2018). As a result, the evidence of damage to seafloor cables provides a scientifically valuable information source for understanding the extent and nature of processes that occur underwater in relation to volcanic eruptions. The extensive damage to seafloor cables following the 2022 eruption of Hunga volcano provided previously unknown insights into not only the long runout (100 s of km) of volcaniclastic density currents triggered by eruption column collapse but also of the remarkable speeds (up to 122 km/h) that can be attained and of the initiation mechanism (i.e. from the collapse of an eruption column into the ocean) based on the extent and timing of cable damage (Clare et al. 2023a).

Examples of cable damage presented in this paper confirm that a significant proportion of volcanic material mobilised by processes such as PDCs, or remobilised as lahars, is transported to the ocean. For example, around 1 × 10^7^ m^3^ of volcanic material (one0 third of the total mobilised volume) transported by the largest PDC during the 1902 eruption of Mount Pelée accumulated offshore (Gueugneau et al. 2020). Given the timing of one of the cable breaks offshore Martinique, it is likely that this offshore flux initiated a volcaniclastic density current, but the ultimate fate and true runout length attained remain unknown. Ocean-entering fluxes of volcanic material and processes that originate in the submarine realm can involve the transport of material across distances that far exceed the scale of volcanic-related processes on land, transporting material into water depths of thousands of metres (Korup 2012). While a volcanic eruption can contribute large volumes of material for near instantaneous mobilisation, deep sea sediment transport related to volcanic eruptions can also involve the remobilisation of material that accumulated long before, such as the submarine slope failures that occurred during the relatively quiet onset of the 1902 eruption of Mount Pelée (Chrétien and Brousse 1989) or those triggered by volcanic lateral collapse, which can lead to flow transformations running out for many tens of kilometres (Watt et al. 2019). Submarine volcaniclastic density currents can also entrain additional material through erosion at their base, and bulk up to create even larger volume flows (e.g. additional 3.5 km^3^ was eroded by density currents on the submerged flanks of Hunga volcano in 2022; Seabrook et al. 2023).

### Which volcanoes should we be most concerned about in relation to subsea cable damage?

While there is clearly value in analysing instances of cable damage associated with past volcanic eruptions, such records provide only a fraction of the possible damage scenarios. They also do not provide direct information about other volcanoes that have the potential to erupt and trigger cable-damaging events. This limits our ability to assess the wider risks posed by volcanic hazards. We therefore now discuss some of the types of scenarios and locations of volcanoes that have the potential to cause damage in the future, based on knowledge gained from cable breaks adjacent to other volcanoes. The greatest risks appear to be from volcanic settings that transport large volumes of volcanic sediment or rock quickly into the ocean and onto steep submarine slopes. The largest eruptions from submarine or subaerial volcanoes (VEI 6) are obvious candidates for transporting vast quantities of volcanic material into the ocean via PDCs and other mechanisms. These events are, however, comparatively rare, with recurrence intervals on the order of hundreds of years (Deligne et al. 2010).

**Fig. 8:**
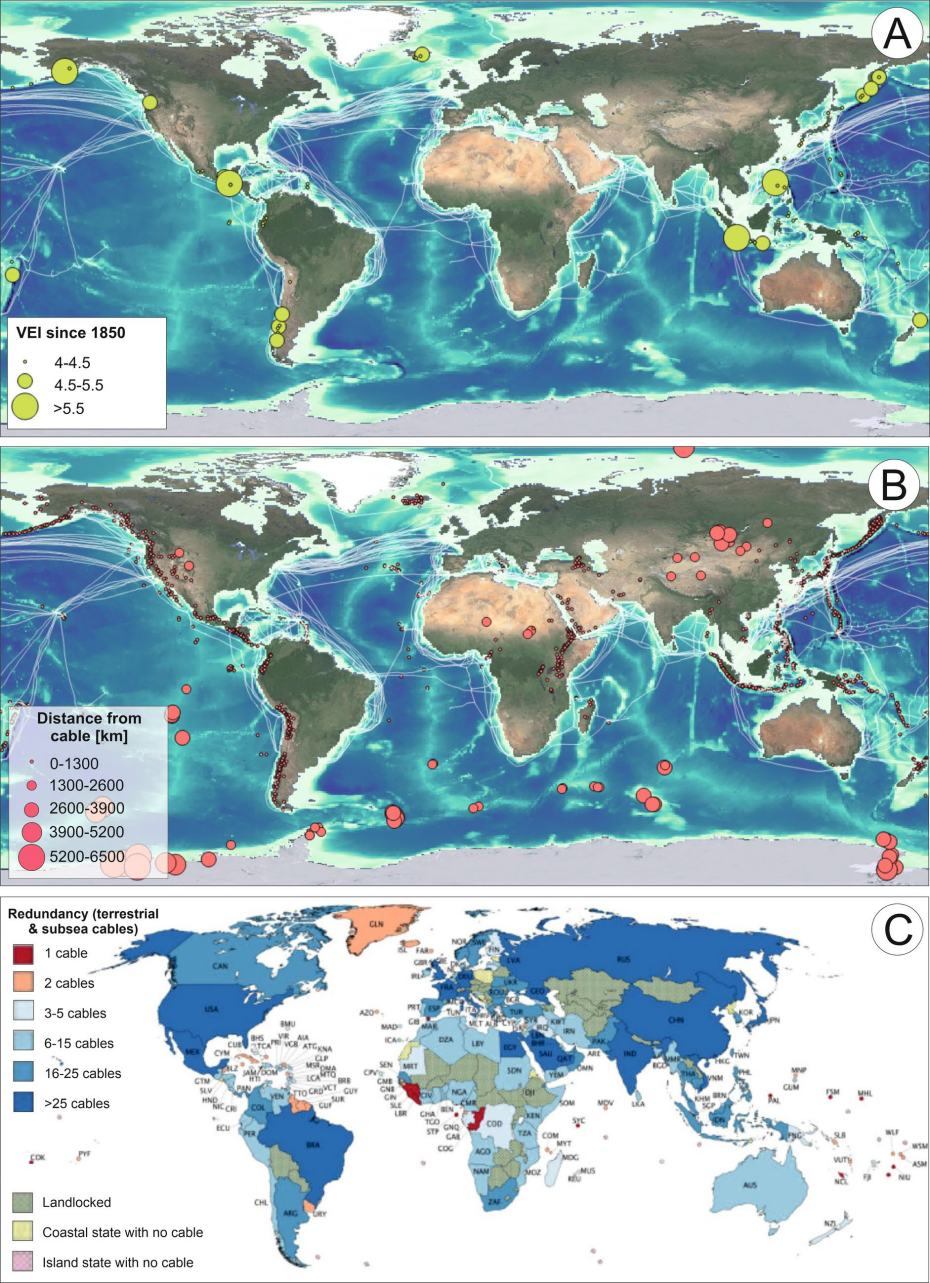
Global context for risks posed by volcanic eruptions to subsea cables, including: reported Volcanic Explosivity Index (VEI) for eruptions recorded worldwide based on GVP (2024) since 1850 (i.e. when subsea telegraph cables were first installed) for eruptions greater than VEI 4 (**A**); distance of mapped volcanoes relative to in-service subsea telecommunications cables based on known volcanoes (**B**); and overview of national redundancy of telecommunications based on number of terrestrial and subsea cable systems (modified from Franken et al. 2022) (**C**). Note that many of the least resilient locations are in volcanically active regions (e.g. South Pacific, Caribbean). Global topography and bathymetry in (**A**) and (**B**) from Smith and Sandwell (1997)

Several large eruptions (VEI > 4) that lie in a similar configuration are reported in the GVP eruption database, but which occurred at a time when there was no nearby subsea cable installed; hence, no cable break will exist in our database (Fig. 8A). However, new cables have been installed close to many of these volcanoes, or are planned for future connections. Therefore, volcanoes such as these are potential candidates for future cable damage. Examples of these include (but should not be limited to) Chaitén (Chile), Rabaul and Manam (Papua New Guinea), Chachadake (Russia), Awu, Ruang and Karangetang (Indonesia), Kuchinoerabujima, Suwanosejima and Hokkaido-Komagatake (Japan), Pinatubo and Mayon (Philippines), Niuafo'ou (Tonga), Stromboli, Vesuvius, and Etna and Campi Flegrei (Italy). The 1991 VEI 6 eruption of Mount Pinatubo was the second largest of the twentieth century and generated long runout PDCs that traveled at least 25 km to reach the coastline and entered the ocean (Pierson 1992). The effects of the 2008/2009 eruption of Chaitén included large-volume lahars which reached the ocean, including some of the greatest sediment yields reported following a volcanic eruption (Major et al. 2016). Such was the volume of sediment supplied that secondary lahars continued to recur several years after the Chaitén eruption, causing major geomorphic modification at the coastline, and presumably further offshore (Major et al. 2016). As demonstrated by the various case studies in this review, these sorts of ocean-entering hazards have the potential to impact and damage subsea cables, and hence should be taken into consideration for future cable routing. Given the limited observations of moderate to large eruptions in historical databases (particularly in regions with limited monitoring), future studies could look at the use of other volcanoes as analogues to infer hazard potential for volcanoes or regions which are less well understood (e.g. Tierz et al. 2019, 2021; Burgos et al. 2023).

A much higher frequency of cable-damaging events can be expected from small–moderate-sized eruption episodes from composite or stratovolcanoes close to a shoreline. Eruptions of these volcanoes may be VEI < 4, but they are among the most numerous and frequently erupting volcanoes in the world. Such eruptions produce PDCs from dome or column collapses, while long eruptions generate potential lahars and enhanced fluvial processes to move large sediment volumes into the ocean. Stratovolcanoes are prone to instability, and any landslides from near-shore or partly submerged ones can generate underwater density currents. Future studies could look to analyse smaller VEI events, as well as identifying regions that have a greater frequency of moderate to high VEI eruptions, where the risk profile would be higher. 

Locations that have few cable connections are generally more vulnerable (Fig. 8C), such as Tonga that has only one international connection; hence, it is important to ensure routes are carefully designed and that appropriate back-up measures (e.g. satellite communications) are in place in case of any outages. However, a greater number of cables does not always guarantee resilience if systems are all laid in a location that could be exposed to the same hazardous event, as was the case for the 1902 eruptions of Mount Pelée (Martinique) and La Soufrière (St Vincent). Several so-called pinchpoints exist globally, where multiple cable systems have to be routed close to each other, typically because of topographic restrictions (e.g. narrow straits or inlet seas), or where multiple cable systems connect to a strategic hub. Several examples exist, where such locations have been affected by extreme natural hazards or accidental human activity that damaged multiple cable systems, with sometimes profound socio-economic impacts. For example, earthquake-triggered sediment density flows in the Luzon Strait (Philippines) created a total of 21 instances of damage on nine out of 11 cables in 2006, reducing Hong Kong’s communications capacity to 20% and halting financial trading in Korea (Rauscher 2010). It took 11 ships (40% of the global repair fleet) 7 weeks to complete the repairs, with the main financial trading centre in Asia reliant on only a single cable (Rauscher 2010). Of greater relevance to volcanic hazards is the location of Hawai’i that provides a key telecommunications hub, with six international fibre-optic cable systems connecting between the mainland USA, Asia, and the South Pacific (Telegeography 2024). While most eruptions on the Island of Hawaii are effusive, explosive phreatomagmatic events occur occasionally, and other hazards such as collapse of submerged slopes (albeit with long recurrence intervals) should be considered (Moore et al. 1994; Dominey-Howes and Goff 2009).

Perhaps the most challenging assessment of risk relates to submarine volcanism in regions where detailed seafloor mapping is sparse or absent. For example, new hydrographic surveys and satellite imagery in the South Pacific are increasingly revealing new seamounts and erupted pumice that forms floating rafts on the sea surface, indicating that some submarine volcanoes may be more active or frequently erupting than previously thought (Bryan et al. 2004; Manga et al. 2018; Nishikawa et al. 2023; Yeo et al. 2024). While we continue to discover new submarine volcanoes, locations expected to feature the most explosive and hazardous eruptions (e.g. volcanic arcs) tend to be mapped at comparatively high resolution (Verolino et al. 2024), but this is not always the case. Detailed route surveys can assist in the identification and avoidance of such volcanoes. Mid-ocean ridges, such as the Mid-Atlantic Ridge, are often unavoidable features for trans-oceanic cable routes. Ridges tend to be less well mapped than arcs, but the associated volcanic hazard is somewhat lower, primarily relating to effusive lava flows of limited runout (e.g. Rubin et al. 2012; Yeo et al. 2012). Instead, the main hazard to subsea cables at the crossing of mid-ocean ridges is abrasion caused by deep ocean currents and the interaction with outcropping bedrock (Carter 2009). The effects of volcanism therefore primarily relate to the irregular seafloor relief created by past eruptions and its effect on steering abyssal currents, rather than an active volcanic process.

## Lessons learned from cable-damaging eruptions and opportunities to address outstanding challenges

We now summarise the key findings gained from the different examples of cable damage and discuss some of the opportunities that exist to increase our understanding and increase resilience for telecommunications.

### Impacts relating to volcanic eruptions can be spatially extensive and long-lasting

While cable damage relating to volcanic eruptions is relatively rare, the case studies presented in this paper reveal that the impacts can be much larger and longer-lived than attributed to human and other natural causes of damage. Repair or reconnection of cables damaged by processes linked to a volcanic eruption can take weeks to decades (e.g. 18 months in Tonga; 25 years in Montserrat), or in some cases projects may be entirely abandoned (e.g. Krakatau). However, where there is sufficient diversity of routes, the resultant impacts on connectivity can be minimal even where tens of kilometres of cable require replacement (e.g. St Vincent, 2021). The aftermath of volcanic eruptions may also pose additional challenges to cable operations for survey, installation, and repair activities (Table 4).

**Table 4:** Other implications of volcanic eruptions for cable repair and survey operations

Hazard/issue	Associated challenges
Lack of geophysical monitoring, hence establishing at what point it is safe to deploy repair vessels requires a judgement call	Exclusion zones, often imposed around volcanoes during and after activity to protect vessels from potential hazards, may make reaching damaged sites logistically challenging (Manley et al. 2020). In the case of Kick ‘em Jenny in 2015, there was a need to wait until such a time that it was deemed safe to repair the cable
Volcanic eruptions can cause substantial bathymetric changes, posing navigational hazards to vessels	Repeat seafloor surveys at Hunga volcano were required to determine new routes for the repaired cables; however, the risk of a crewed vessel close to the recently erupted volcano was deemed too high. Surveys performed 3 months after the eruption therefore included use of an autonomous surface vessel to map the seafloor adjacent to and within the caldera (Seabrook et al. 2023)
Damage to ports, coastal infrastructure and/or ships from ash fall, pyroclastic density currents, lava flows, lahars, or related slope failure or tsunamis	Repair vessels may need to travel longer distances to reach site or from which to mobilise
Rafts of floating pumice can block vessel water intakes, abrade hulls and endanger operations	Pumice rafts may cover thousands of square kilometres of the ocean, hampering efforts to reach repair sites or carry out repairs once there (Wilson et al. 2014; Cragg et al. 2024). Satellite-based mapping and surface ocean current may assist in determining location and movement of rafts (Carey et al. 2014)
Limited or loss of communications affecting coordination for repairs	Access can be further complicated where damage to cables results in loss of communications, which may be even greater during large magnitude eruptions

### Network diversity and redundancy can enhance telecommunications resilience

Additional and more geographically diverse cable routes and landing stations can provide greater resilience or better contingency in case of damage to subsea cable systems; however, some of the most at-risk countries have very few cable connections (e.g. Tonga, Vanuatu, Kiribati; Papua New Guinea; Franken et al. 2022; Watson 2022). Identifying alternative appropriate route options is challenging in regions where there is sparse detailed bathymetric data, particularly those that are geologically complex like the Tonga–Tofua Arc. There, steep slopes lie to the east and north of the islands of Tonga where they transition to a deep-sea trench, which is a focal point for seismogenic earthquakes. The steep slopes can also be prone to slope failure and are incised by submarine canyons; and hence, these are suboptimal locations for cable routing and do not really represent a viable lower-risk solution. Building redundancy into subsea networks is routine in many parts of the world; however, a key issue for small islands, such as those in the South Pacific, is securing the financial backing for systems that connect relatively small populations where there is not a huge demand for bandwidth (Internet Society, 2017; Kaul et al. 2024).

One of the biggest challenges facing the repair operation offshore Tonga in 2022 was the vast extent of damage, requiring significant lengths of new cable (Clare et al. 2023a). Holding a more local stock of replacement cable could mitigate this in future; however, the damage caused by the 2022 eruption was unprecedented and such stock-piling would require assessment on a cost–benefit basis. Regardless, investment in other technologies that can provide an emergency back-up is particularly important for remote island communities. Low-level satellite and microwave networks (e.g. where there is line of sight to another unaffected island; Arai, 2018; Digwatch 2020) can fill part of that gap. While they may only account for a small percentage of the cable-carrying data traffic, these other technologies can assist with rapidly reconnecting global communications, enabling local disaster response and coordinating aid relief efforts, as was the case following the 2011 Haiti earthquake that destroyed the only cable landing station as well as the police land mobile radio system (Internet Society, 2017). A WiFi bridge was established with the neighbouring Dominican Republic to reconnect Haiti within 48 h of the earthquake (Internet Society, 2017). The need for back-up scenarios is particularly important as many of the locations that are most exposed to volcanic hazards (e.g. volcanic islands) are those that are also exposed to a variety of other natural hazards such as sea level rise, storm surges, and seismicity. People living in these locations are disproportionately reliant on subsea cables, due to their remoteness, for receiving funds from abroad, tourism, e-commerce, telemedicine, online education, and other services to enable sustainable development and economic growth. Future research, in collaboration between academia, the subsea cable industry, and regional and local stakeholders, will help to better understand risks and enhance the resilience of critical seafloor connections that are fundamental to small islands and coastal communities worldwide.

### Subsea cables provide opportunities to fill gaps in monitoring and early warning of volcanic hazards worldwide

Many remote oceanic regions are poorly covered by seismic networks, including the South Pacific, creating a relative blind spot with respect to early warning of volcanic hazards (Goff and Terry, 2016). Even where subsea monitoring is performed, the complexity of the seafloor and positioning of sensor arrays such as hydrophones means that any signals are also often poorly recorded. Volcanoes typically do not generate large magnitude earthquakes that can be detected by global seismic networks, particularly in the unrest phase, which is why on land most volcanoes are instrumented to record low magnitude signals that may indicate a future event. This is rarely the case in the oceans, meaning that many hazardous submarine volcanoes remain un- or poorly monitored. Almost none has dedicated long-term monitoring systems, with a few exceptions (e.g. SANTORY at Kolumbo, Ionian Sea and offshore Mayotte, Indian Ocean; Nomikou et al. 2022; Aiken 2024). While there is nothing that can be done to prevent an eruption, a better understanding of the states of activity of submarine volcanic edifices provided by seismic information and repeated seafloor mapping could be used to provide a greater understanding of the hazards these volcanoes pose to communities and infrastructure.

Opportunities for monitoring volcanic hazards exist with respect to the design of new cable systems and the use of existing ones. Recent advances in technology now enable the use of the optical fibres at the core of modern telecommunications cables as a sensing tool to make measurements of temperature, strain, seismicity, and a variety of volcano-tectonic processes. Approaches such as distributed acoustic sensing that analyse backscattered light along an optical fibre can effectively turn a telecommunications cable into a series of seismometers or hydrophones without any requirement for modification, enabling 1-m spatial resolution along distances of up to around 150 km from the shore (Lindsey and Martin 2021). Prior to its repair in 2023, distributed acoustic sensing was performed along the unbroken part of the domestic cable that connected the main island of Tongatapu to the island groups of Tonga to the north (Nakano et al. 2024). During only 7 days of monitoring, 17 small magnitude earthquakes were recorded by the cable, including an event located beneath Hunga volcano itself, demonstrating the utility of distributed acoustic sensing to provide low-cost, real-time offshore hazards monitoring in a region that has very limited land-based seismic stations (Nakano et al. 2024). While there has been limited application to monitoring subsea volcanoes, the efficacy of distributed acoustic sensing has been demonstrated on terrestrial networks. For example, the timing and location of volcanic earthquakes were characterised along a terrestrial cable near Azuma volcano, Japan (Nishimura et al. 2021). Strain signals associated with volcanic explosions of Mount Etna were detected using distributed acoustic sensing along a fibre-optic cable (Jousset et al. 2022). This approach also detected very small volcanic events, related to fluid migration and degassing that show promise for the use of distributed acoustic sensing as an early warning system to detect precursor events before larger eruptions, which has been demonstrated using a land-based fibre-optic cable during an eruption in Grindavík, Iceland (Li et al. 2025). There remains a need to better understand how effectively this may be applied in the submarine realm, particularly as ineffective seafloor coupling can lead to degraded signal to noise ratio (e.g. as shown offshore Santorini; Igel et al. 2024).

Other methods of fibre-optic sensing have been shown to be capable of detecting and localising a range of natural processes at high sensitivity, including seismicity along subsea cables. Interferometric monitoring makes use of an ultra-stable laser at one end of a cable and detects changes in the phase of the returned light signal that are created by environmental perturbations. The initial application of interferometry along a subsea cable made an integrated measurement along the full cable length to detect earthquakes (Marra et al. 2018); however, recent developments make use of a circuit in each of the repeaters that are used to boost the optical signal along its course. It is now possible to use interferometry, and another similar approach that detects changes in the state of polarisation of returned light, to monitor at a spatial resolution equivalent to the spacing between individual repeaters (typically tens of kilometres apart) and to detect a range of processes, which include volcanic tremors, microseismicity, and seafloor fluid flow (Marra et al. 2022; Zhan et al. 2021). As fibre-optic cable sensing can be performed remotely, and does not require maintenance of sensors, these fibre-optic-based observing approaches may be highly suitable for monitoring other volcanoes and could enable a greater forewarning of events, and information on when an event has ended, providing constraints on when it is safe to return after an evacuation. A key benefit of these approaches is that they do not require any physical modification, and so can be used on existing data carrying cables without any interruption of telecommunications, providing an exciting opportunity to create new monitoring networks to fill key geographic and knowledge gaps.

Opportunities for new systems that involve bespoke hybrid cable designs include the Science Monitoring And Reliable Telecommunications (SMART) cable initiative (Howe et al. 2022). A SMART cable is a modified version of a commercial telecommunications cable, which includes a specially designed repeater unit or node that integrates scientific sensors to monitor seismicity, tsunamis, and other environmental information, as well as the standard industry equipment (Howe et al. 2022). As a SMART cable requires a bespoke design, this approach cannot be retrofitted to existing systems. The first deployment in a volcanically active region is likely to be installed between New Caledonia and Vanuatu, with the contract signed in 2024 (Capacity Media 2024), which will provide new capability for the region. 

## Conclusions

Damage to subsea cables related to volcanic eruptions is rare (< 1% of all instances of cable damage globally), but when this happens it can have major impacts; cutting off entire countries from global communications, creating far greater damage than other natural processes and human activities, with long-lasting effects (months to decades), particularly affecting vulnerable small island communities. A review of instances of damage associated with volcanic eruptions from multiple sites worldwide reveals that the processes that cause damage can be extremely variable and are mostly due to cascades of hazards, particularly that initiate mass movement events that enter the ocean (e.g. lahars or PDCs) or initiate submarine landslides. While there is no apparent VEI threshold for cable-damaging events, cable damage associated with volcanic eruptions identified in this study is related primarily to explosive eruptions, with larger eruptions causing the greatest damage. Steep-flanked coastal or island volcanoes, or shallow/partially submerged explosive volcanoes appear to pose the greatest threat. Planning of cable routes using detailed surveys can help to minimise any potential risk, but in many cases, avoidance is not possible (e.g. where a cable is necessary to connect to a volcanic island). The first course of mitigation is therefore to ensure that there is sufficient redundancy in a regional network, which can be improved by ensuring geographic diversity in routes and landing points; however, many small islands have a few or only a single cable connection and it can be challenging to secure financial investment due to the limited demand for bandwidth. In such scenarios, where there is limited diversity of routes, then investment in back-up solutions, such as low-level satellite coverage, and holding sufficient lengths of spare cable in store are the next best approaches to ensure that some level of communications can continue, which is particularly important in the midst of a volcanic crisis. This first global study of volcanic hazards for subsea cables not only provides considerations that can assist in ensuring that communications remain as resilient as possible, but also reveals how instances of cable damage can provide valuable insights into the submarine components of explosive volcanic eruptions, which often go unrecognised. Advances in technology are providing new opportunities to use the optical fibre at the core of existing telecommunications cables not only to monitor the health of a subsea cable, but also as a monitoring tool that has potential to enable the early warning of volcanic hazards and fill existing hazard blind spots for the benefit of small island and coastal communities and will provide important new insights into the poorly observed behaviour of submarine volcanoes.

## Supplementary Information

Below is the link to the electronic supplementary material.

Supplementary file1 (DOCX 67 KB)
