# Supplementary material for: Volcanic eruptions and the global subsea telecommunications network
Source: Bull Volcanol. 2025 Jun 4;87(6):51. doi: 10.1007/s00445-025-01832-1 (PMC12133983; doi:10.1007/s00445-025-01832-1)
Supplement: Supplementary file 1 — Supplementary file1 (DOCX 67 KB) [file 445_2025_1832_MOESM1_ESM.docx]

**Table S-1: Volcanoes mentioned in this study other than those in the specific case studies, with reference to examples of infrastructure damage.**

| **Volcano** | **Country** | **Eruption year** | **Examples of infrastructure damage** | **VEI** | **Reference** |
| --- | --- | --- | --- | --- | --- |
| Pinatubo | Philippines | 1991 | Widespread damage to irrigation and flood control systems, roads and bridges and school buildings. Damage primarily from pyroclastic density currents, lahars and ash fall. | 6 | Tayag and Punongbayam (1994) |
| Crater Peak | Alaska, USA | 1992 | Increases in turbidity in water treatment due to ash fall and HVAC intakes for the telephone exchange were blocked leading to shutdown | 3 | Wilson et al. (2014) |
| Chaitén | Chile | 2008 | Damage to houses, infrastructure and port by long runout lahars that caused avulsion of river to breach through town | 4 | Pierson et al. (2013) |
| Ruapehu | New Zealand | 1995/96 | 215 km-runout lahar that had little damage to infrastructure due to effective planning and warning systems. | 3 | Massey et al. (2010) |
| Kasatochi | Alaska, USA | 2008 | Remote volcanic eruption with limited impacts, but the ash and gas cloud interfered with air travel between Alaska and conterminous USA causing at least 40 flight cancellations | 4 | Waythomas et al. (2010) |
| Okmok | Alaska, USA | 2008 | Widespread ashfall but remote eruption with limited impacts on infrastructure. | 4 | Unema et al. (2016) |
| Eyjafjallajokull | Iceland | 2010 | Ash cloud had major impacts on aviation traffic, with large scale closure of European and Trans-Atlantic airspace. | 4 | Harris et al. (2012) |
| Nabro | Eritrea | 2011 | Major SO2 emissions with impacts on human health. Seismicity, lava flows and ash fall damaged housing and farm buildings. | 4 | Goitom et al. (2015) |
| Grimsvotn | Iceland | 2011 | Most economic losses suffered by farmers due to damage to metal cladding, window panes, walls and floors. | 4 | Gumundsson et al. (2021) |
| Sinabung | Indonesia | 2013 & 2019 | Lava flows, pyroclastic density currents and ash fall damaged houses and agricultural land. | 4 | Utami et al. (2021) |
| Semeru | Indonesia | 2014 & 2017 | Pyroclastic density currents and ash fall damage wide range of infrastructure. Post-eruptive lahars devastated villages and towns. | 4 | Thouret et al. (2023) |
| Manam | Papua New Guinea | 2004 | Large scale evacuations with long term impacts to agriculture, settlement infrastructure and water supplies. | 4 | Cotterill et al. (2024) |
| Kelud | Indonesia | 2014 | Ash fall led to severe damage to houses. Lahars damaged buidings 35 km from the vent. | 4 | Williams et al. (2020) |
| Calbuco | Chile | 2015 | Ash fall led to collapse of buildings. 26 houses swept away by lahars. | 4 | Hayes et al. (2019) |
| Wolf | Galapagos, Ecuador | 2015 | Remote volcanic eruption with negligible impacts. | 4 | Smithsonian (2015) |
| Ulawun | Papua New Guinea | 2019 | Lava flows and pyroclastic density currents forced around 11,000 people flee their homes. Heavy ash fall damaged several main roads, houses and schools. | 4 | Orynbaikyzy et al. (2023) |
| Taal | Philippines | 2020 | Major damage to roads due to ashfall and disruption to water supplies. | 4 | Santos et al. (2023) |
| Fukutoku-Oka-no-Ba | Japan | 2021 | Pumice raft caused damage to coastal infrastructure such as ports and harbours in Okinawa. | 4 | Maeno et al. (2022) |

**Table S-2: Key events in the 1883 eruption of Krakatau, synthesised from accounts in Sturdy (1884), Symons et al. (1888), Latter (1981), Simkin and Fiske (1983), Self, (1992), Dörries (2003), Winchester (2003), Madden-Nadeau et al. (2021) and Toivanen (2021).**

| **Date and time** | **Observations** |
| --- | --- |
| 20^th^ May 1883 | Eruption commenced with Vulcanian to sub-Plinian activity, creating a 20 km-high eruption column |
| 23^rd^ May 1883 | Volcanic activity subsiding, with only low levels of activity observed for the next month |
| 24^th^ June 1883 | Low-level eruptions, earthquakes and occasional violent explosions commenced, continuing throughout July |
| 26^th^ August 1883 | Eruption escalated, switching to a Plinian mode of activity. A 26 km-high eruption column had been formed by 14:00, with volcanic explosions occurring every ten minutes. |
| 26^th^ August 1883 - 14:00 | A telegraph message was sent along the subsea cable network from Batavia, providing the first reports of the eruption that reached Europe within a day. This was rapidly reported upon by newspapers worldwide |
| 26^th^ August 1883 - 15:30-17:00 | Explosions were heard 670 km away and across the entirety of Java by 17:00, with the first explosion recorded on a barometer at the Batavia gas works shortly after 15:30. |
| 26^th^ August 1883 - 18:00 | Subsea telegraph cable that linked Anjer and Teluk Betung to the north was cut during a telegraphic message from the Anjer Telegraph Master (Schruit), noting unusual darkness and the ongoing eruption. |
| 26^th^ August 1883 - 18:10 | First tsunami - recorded in Batavia at 18:10 |
| 26^th^ August 1883 - 19:30 | A second telegraph cable from Anjer, to Merak on the Javan coast north of Anjer, remained active. Reports at 19:30 from Merak of telegrams describing the situation in Anjer suggest that information continued to be sent along the coast through the evening. Schruit’s report suggests that the telegraph cable had been snapped close to shore by the mast of a schooner or other debris from multiple smaller vessels that had been sunk in the harbour area by vigorous tsunami waves. |
| 26^th^ August 1883 - 01:00 | Arrangements were made for repair of the broken telegraph cable, which commenced at 01:00 (Toivanen, 2021) but were ultimately unsuccessful. |
| 27^th^ August 1883 - 10:00 | Eruption reaches its climax, with the largest tsunami occurring coincident with the most powerful explosion; inundating shorelines around the Sunda Strait and drowning tens of thousands. 80 km high eruption column. |

**Table S-3: Key events in the 1902 eruption of Mount Pelée, synthesised from accounts in Chrétien and Brousse (1989), Westercamp (1987), Chrétien (1983), Tanguy (1994) and Gueugneau et al., (2020).**

| **Date and time** | **Observations** |
| --- | --- |
| 22^nd^-23^rd^ April 1902 | Small phreatic eruptions and up to 4-5 M_w_ earthquakes, with low level phreatic activity occurring until 5^th^ May.  Subsea telegraph cable damage on 22^nd^ April. |
| 25^th^ April 1902 – 07:00 | Short-lived explosions noted in the summit region on 25^th^ April at 07:00, coincident with 4-5 M_w_ earthquakes. |
| 25^th^ April 1902 – 09:00 | At 09:00 on the same day, around 500,000 m^3^ of ash was ejected during a larger phreatic eruption. |
| 25^th^ April 1902 | River discharge increased from the 25^th^ April in response to elevated rainfall; first in the Rivière Blanche, then Rivière due Precheur, and in all rivers from the 6^th^ May. |
| 26^th^ April 1902 | A period of relative calm ensued between 26^th^ April and the 1^st^ May. |
| 2^nd^ May 1902 | On 2^nd^ May, earthquakes were felt, and large explosions occurred on 3^rd^ and 4^th^ May. |
| 3^rd^-6^th^ May 1902 | Four subsea telegraph cables damaged where lahars entered the ocean. |
| 5^th^ May 1902 | On the 5^th^ May and between 7-8^th^ May, destructive lahars occurred, which took at least 400 lives and isolated the village of La Prêcheur. |
| 6^th^ May 1902 | Phreatic eruptions intensified on the southern flank on 6^th^ May |
| 7^th^ May 1902 (evening) | First pyroclastic density currents ran down the Rivière Blanche valley. |
| 8^th^ May 1902 – 06:00-08:00 | The eruption escalated to its climax on 8^th^ May, when jets were emitted from the caldera between 06:00 and 08:00, before a supersonic shock wave was generated by a VEI 4 eruption that triggered a pyroclastic density current.  One subsea telegraph cable damaged. |

**Table S-4: Key events in the 1902 eruption of La Soufrière, synthesised from accounts in Huggins (1902), Anderson and Flett (1903), Smith and Roobol (1975) and Cox (2004).**

| **Date and time** | **Observations** |
| --- | --- |
| February – April 1902 | Period of unusually-elevated seismicity. |
| 5^th^ May 1902 | Lake level changes and phreatic activity noted at La Soufrière volcano |
| 5^th^-7^th^ May | Five subsea telegraph cables damaged. |
| 6^th^ May 1902 – 15:00 | The settlements of Wallibou and Richmond were evacuated following a cloud of steam that burst was emitted from the crater |
| 7^th^ May 1902 (morning) | Explosions led to ash fall and further evacuations, ahead of the VEI 4 eruption climax in the afternoon, during which 1680 people died.  Powerful pyroclastic density currents and lahars entered the ocean, including at the outflow of the Wallibou River and down multiple catchments.  Subsea telegraph cable to St Lucia broke 5 minutes after pyroclastic density current entered the ocean. |

**Table S-5: Key events in the 1995-97 eruption of Soufrière Hills volcano, synthesised from accounts in Aspinall et al. (1998), Cole et al. (1998), Jackson et al. (1998), Young et al. (1998), Edmonds and Herd (2005), Herd et al. (2005) and Trofimovs et al. (2006)**

| **Date and time** | **Observations** |
| --- | --- |
| 18^th^ July 1995 | Eruptive activity commenced as release of steam via multiple vents, which later coalesced. |
| Late July 1995 | Phreatic explosions occurred intermittently over a period of four months, with associated ash columns that reached up to 3 km height and regular volcano-tectonic earthquakes. |
| August 1995 | Evacuation of Plymouth (the island capital). |
| Late September 1995 | A swarm of earthquakes occurred on the western flank of the volcano, immediately before the growth of a 40,000 m^3^ dome. |
| Mid November 1995 | Start of dome extrusion and continued growth (up to 30 m/day vertical growth) with associated seismicity and occasional collapse. |
| Early March – September 1996 | Dome growth became focused on the north-eastern flank, which created a larger collapse and the first pyroclastic density currents that reached the coast, followed by similar events in May, July, August and September; depositing several million cubic metres of material. |
| 17^th^ September 1996 | A major collapse of 40% of the dome depressurised the dome interior, resulting in a 14 km-high eruption column. |
| 11^th^ December 1996 | More rapid dome growth, leading to intense dome collapse and associated pyroclastic density currents, also focused within the Tar Valley to the east |
| Late March 1997 | Collapses triggered 4 km-runout pyroclastic density currents in the White Valley to the south-west and inundating Plymouth before entering the ocean. |
| Late September 1997 | The biggest collapse (85 x 10^6^ m^3^) occurred during a period of enhanced explosive activity.  Cable landing station in Plymouth destroyed. |
| October 2020 | New fibre optic cable connected to north-east of island to provide higher capacity telecommunications . |

**Table S-6: Key events in the 2021-22 eruption of Hunga volcano, synthesised from accounts in Brenna et al. (2022), Lynett et al. (2022), Le Mével et al. (2023), Millan et al. (2022), Wright et al. (2022) and Seabrook et al. (2023).**

| **Date and time** | **Observations** |
| --- | --- |
| 19^th^ December 2021 | Two explosive eruptions on 19^th^ December created steam-rich gas and ash plumes that reached into the troposphere. |
| 15^th^ January 2022 | Explosive eruptions continued until the 15^th^ January, when the episode reached its climax. An explosion destroyed the cone, removing the connection between the two islands and leaving only small fragments of them behind. |
| 15^th^ January 2022 – 03:47 | The start of the climactic eruptive phase was marked by a low eruptive plume, that rose to a height of >10 km. |
| 15^th^ January 2022 – 04:15 | A VEI 6 explosion occurred. |
| 15^th^ January 2022 – 04:17-04:20 | Growth of umbrella-shaped eruption plume that reached around 18 km height. Plume collapses into the ocean initiated, and continued beyond 04:20 due to the high eruption rates. |
| 15^th^ January 2022 – 04:21-04:25 | A second explosion occurred, with further explosions occurring until 04:25, when a pressure wave initiated that was felt around the world. |
| 15^th^ January 2022 – 04:30 | Damage to domestic fibre-optic cable due to fast-moving volcaniclastic density current triggered from column collapse. |
| 15^th^ January 2022 – 05:44 | Damage to international fibre-optic cable by volcaniclastic density current. Tonga cut off from global telecommunications. |
| 22^nd^ February 2022 | International fibre-optic cable repaired. |
| 12^th^ July 2023 | Domestic fibre-optic cable repaired. |
